# Targeting miR-21 ameliorates colitis by restoring Th17/Treg balance through direct inhibition of Smad7/Ski

**DOI:** 10.1016/j.omtn.2026.103120

**Published:** 2026-09-29

**Authors:** Yue Wen, Xia Yi, Xiaowen Zhu, Han Wang, Dean Tian, Ge Wang

**Affiliations:** 1 Department of Gastroenterology, Tongji Hospital, Tongji Medical College, Huazhong University of Science and Technology, Wuhan, Hubei, China; 2 Hunan Provincial University Key Laboratory of the Fundamental and Clinical Research on Neurodegenerative Diseases, Changsha Medical University, Changsha, Hunan, China; 3 Department of Gastroenterology, Taihe Hospital, Hubei University of Medicine, Shiyan, Hubei, China

**Keywords:** MT: Non-coding RNAs, miR-21, ulcerative colitis, Smad7, Ski, Th17/Treg balance, CD4^+^ T cell differentiation

## Abstract

MicroRNA-21 (miR-21) has been implicated in ulcerative colitis (UC) pathogenesis and is hypothesized to participate in disease progression by regulating CD4^+^ T cell differentiation, although the precise molecular mechanisms remain unclear. Using clinical samples and a dextran sulfate sodium-induced colitis mouse model, we found miR-21 significantly upregulated in inflamed tissues. In mice, upregulation of miR-21 promoted differentiation of proinflammatory T helper (Th)2 and Th17 cells while suppressing anti-inflammatory regulatory T (Treg) cells. We identified Smad7 and Ski, two inhibitors of the TGF-β pathway, as direct targets of miR-21. By repressing these targets, miR-21 activates the phospho-Smad2/3 pathway, thereby driving the Th17/Treg imbalance. Therapeutic intervention with miR-21 antagomir in dextran sulfate sodium-treated mice effectively reduced miR-21 levels, restored Smad7 and Ski expression, inhibited phospho-Smad2/3 activation, corrected the T cell imbalance, and alleviated colitis symptoms. Our findings extend the previously established miR-21-Smad7 regulatory model by identifying Ski as a complementary target through which miR-21 amplifies TGF-β signaling and promotes Th17/Treg imbalance. These results provide new mechanistic insight into miRNA-mediated immune dysregulation and suggest the miR-21-Smad7/Ski axis as a potential therapeutic target, especially in UC.

## Graphical abstract

**Figure:**
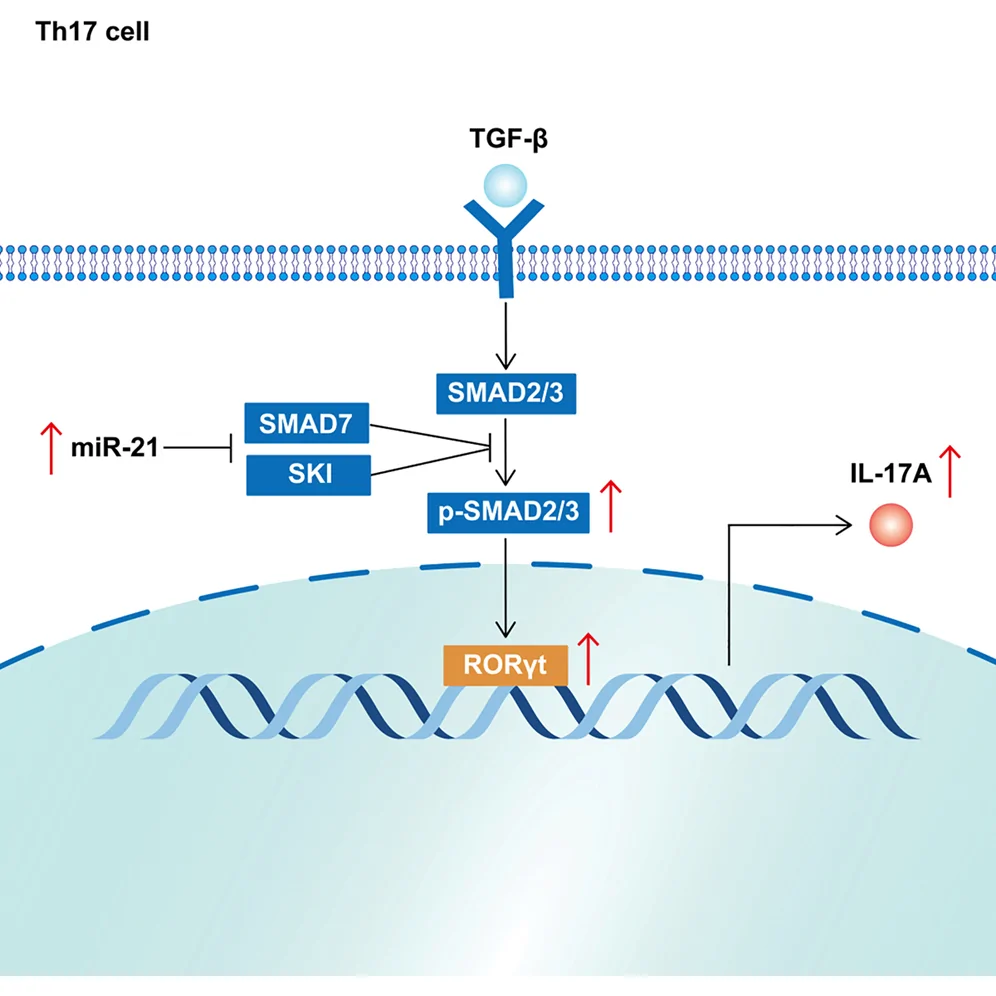

## Teaser

Wang and colleagues identify that microRNA-21 drives inflammatory bowel disease by disrupting the balance between proinflammatory Th17 and regulatory T cells. miR-21 directly targets Smad7 and Ski, activating a key signaling pathway. This work reveals a new therapeutic target, suggesting that inhibiting miR-21 alleviates colitis in mice.

## Introduction

Inflammatory bowel disease (IBD), including Crohn’s disease (CD) and ulcerative colitis (UC), is a chronic, immune-mediated gastrointestinal disorder with a rising global incidence.^1^^,^^2^^,^^3^^,^^4^ Current therapeutic strategies focus on immunomodulation, cytokine-targeting biologics, and gut microbiota intervention.^5^ However, a significant proportion of patients experience primary non-response or secondary loss of response to these treatments, underscoring the urgent need to explore disease mechanisms for the development of novel prevention and treatment strategies.

The pathogenesis of IBD involves complex interactions among genetic susceptibility, environmental factors, and dysregulated immune responses.^6^^,^^7^ A critical aspect of this dysregulation is the imbalance in CD4^+^ T helper (Th) cell subsets, where CD is associated with Th1 responses and UC with an atypical Th2 profile. Recent research further confirms the critical role of Th17/regulatory T (Treg) cell imbalance in IBD pathogenesis.^8^

MicroRNAs (miRNAs) are key post-transcriptional regulators of gene expression and immune homeostasis,^9^^,^^10^^,^^11^^,^^12^ highlighting their potential as biomarkers and therapeutic targets.^13^^,^^14^^,^^15^^,^^16^ Among them, miR-21 is notably dysregulated in IBD, although its reported expression levels and functional roles are conflicting. While often elevated in active UC and pediatric CD tissues, some studies report decreased levels in peripheral blood.^14^^,^^17^^,^^18^^,^^19^^,^^20^ Similarly, in animal models, miR-21 can exert both pro- and anti-inflammatory effects depending on the context, suggesting a complex, dual role in disease progression.^21^^,^^22^

Notably, miR-21 is significantly upregulated in activated T cells and is implicated in modulating T cell activation and apoptosis.^23^^,^^24^^,^^25^^,^^26^^,^^27^ Clinical research revealed higher miR-21 expression in CD3^+^ T cells from patients with active UC than in those from patients in remission,^28^ strongly implicating miR-21 in IBD pathology through T cell regulation. Accumulating evidence indicates that miR-21 is a key regulator of CD4^+^ T cell differentiation. It has been demonstrated that miR-21 promotes Th17 cell differentiation by targeting Smad7, a negative regulator of transforming growth factor-β (TGF-β) signaling, and mediates pathogenicity in experimental autoimmune encephalomyelitis (EAE).^29^ In multiple myeloma, Rossi et al. further demonstrated that early inhibition of miR-21 in naive T cells impairs Th17 differentiation and abrogates Th17-mediated tumor-promoting functions, including cell proliferation and osteoclast activity.^30^ Moreover, in other disease models, miR-21 has been shown to influence Th17/Treg homeostasis and Treg cell function through the regulation of Foxo1, Skp2, or other molecules.^31^^,^^32^^,^^33^^,^^34^ However, in the context of IBD, it remains unclear whether miR-21 influences disease progression by affecting CD4^+^ T cell differentiation, which target gene networks are involved (particularly novel targets beyond Smad7), and what specific regulatory mechanisms operate during intestinal inflammation.

To address these gaps, this study systematically evaluated the expression characteristics of miR-21 in inflammatory colon tissue by analyzing clinical IBD samples and a dextran sulfate sodium (DSS)-induced colitis model. The present study focuses on UC-like inflammation using a DSS-induced acute colitis model. Furthermore, naive CD4^+^ T cells isolated from mouse spleens via magnetic bead sorting will undergo intervention with miR-21 inhibitors/mimics to observe the regulatory effects on Th1/Th2/Th17/Treg differentiation. This research aims to elucidate the target gene network of miR-21 and its molecular mechanism in regulating CD4^+^ T cell differentiation and to explore the feasibility of targeting miR-21 for the treatment of DSS-induced colitis. These findings provide new insights for biomarker and precision therapy development in UC and UC-like colitis.

## Results

### Upregulation of miR-21 expression in colonic inflammation

To investigate alterations in miR-21 expression in the intestinal mucosa of patients with IBD, mucosal specimens were collected from intestinal polyp controls and confirmed patients with UC and CD at Tongji Hospital, Tongji Medical College, Huazhong University of Science and Technology. On the basis of endoscopic activity grading, patients with IBD were stratified into the following groups: UC remission (*n* = 3) and mild (*n* = 8), moderate (*n* = 6), or severe (*n* = 5) active UC and CD remission (*n* = 6) and mild (*n* = 4), moderate (*n* = 5), or severe (*n* = 4) active CD. Normal intestinal mucosa obtained from patients with colorectal polyps (*n* = 10) served as the control group. RT-qPCR analysis revealed significantly higher miR-21 expression in the intestinal mucosa of patients with active UC than in that of healthy controls, with expression levels being higher in patients with active UC than in those with active CD (Figure 1A). Although the small sample size in the subgroup limited statistical power and generalizability, miR-21 expression exhibited a strong positive correlation with endoscopic disease activity, with higher endoscopic grades associated with increased miR-21 levels (Figures 1B and 1C).

**Figure 1:**
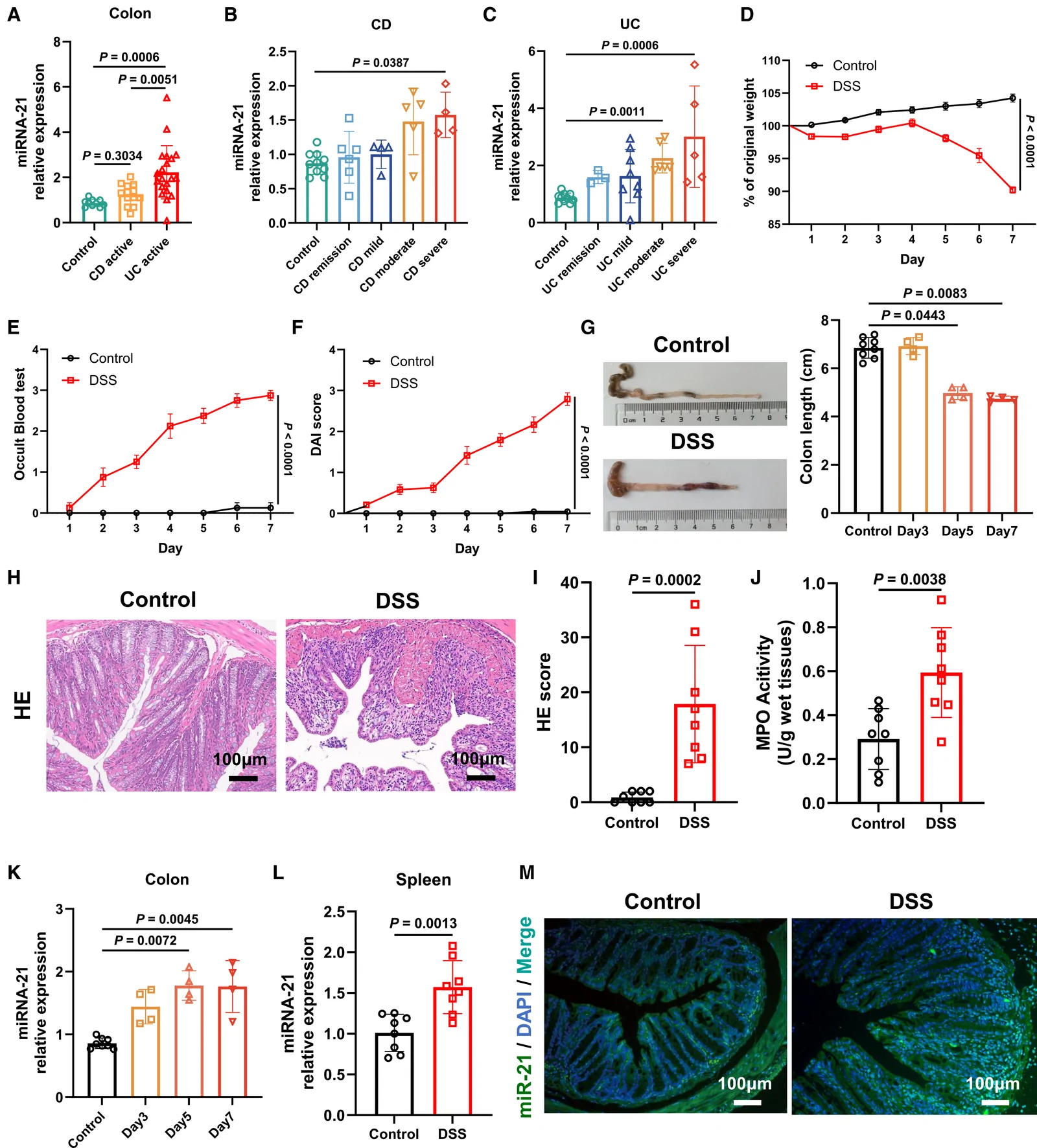
Upregulation of miR-21 expression in colonic inflammation (A) Comparison of miR-21 levels in the intestinal mucosa of patients with active-phase CD and UC vs. intestinal polyp controls. (B) miR-21 levels in CD patient mucosa stratified by endoscopic activity grade vs. controls. (C) miR-21 levels in UC patient mucosa stratified by endoscopic activity grade vs. controls. (D) Percentage change in the body weight of the mice (*n* = 8). (E) Fecal occult blood levels in mice (*n* = 8). (F) Disease activity index (DAI) scores (*n* = 8). (G) Macroscopic appearance of the colons and quantification of colon length (*n* = 8 or 4). (H) Representative H&E-stained images of colon sections (20× magnification). Scale bar: 100 μm. (I) Statistical analysis of histopathological (H&E) scores (*n* = 8). (J) Myeloperoxidase (MPO) activity in mouse colon tissues (*n* = 8). (K) miR-21 expression levels in mouse colon tissues on days 0, 3, 5, and 7 (*n* = 8 or 4). (L) miR-21 expression levels in mouse splenic lymphocytes (*n* = 8). (M) FISH analysis of miR-21 localization and expression in mouse colon tissues (20× magnification). Green: miR-21 probe signal; blue: DAPI-stained nuclei. Scale bar: 100 μm. Data in (A) were analyzed using one-way ANOVA followed by Tukey’s post hoc test. Data in (B), (C), (G), and (K) were analyzed using the Kruskal-Wallis test followed by Dunn’s multiple comparisons post test. Data in (D)–(F) were analyzed using unpaired Student’s *t* tests with Bonferroni correction. Data in (I) were analyzed using the Mann-Whitney U test. Data in (J) and (L) were analyzed using the unpaired Student’s *t* test. Data are presented as the mean ± SD. See also Figure S1.

Additionally, an acute colitis model was established using DSS in male C57BL/6 mice (*n* = 20, 6–8 weeks old). Compared with the normal water-treated group (*n* = 8), DSS-treated mice (*n* = 12, 3% DSS) exhibited weight loss, diarrhea, mucoid bloody stools, and colon shortening, accompanied by significantly elevated disease activity index (DAI) scores (Figures 1D–1G). Histopathological analysis (H&E staining) confirmed neutrophil infiltration, goblet cell disorganization, and crypt destruction in the colonic mucosa of DSS-treated mice (Figures 1H and 1I), with increased histopathological scores and myeloperoxidase (MPO) activity (Figure 1J), confirming successful model induction. Compared with controls, the expression of miR-21 in DSS-induced colons increased over the course of DSS treatment (days 3, 5, and 7 vs. day 0 as a control) and was significantly upregulated in splenic lymphocytes compared to controls (Figures 1K and 1L). Concurrently, the PDCD4 protein (a known target of miR-21^24^) was downregulated in DSS-treated colons (Figures S1A and S1B). Fluorescence *in situ* hybridization (FISH) localized enhanced miR-21 signals predominantly to lamina propria lymphocytes (LPLs) and inflammatory infiltrates in DSS-treated colons, which were associated with the severity of mucosal damage (Figure 1M).

### Altered CD4^+^ T cell subset proportions in DSS-induced acute colitis mice

During intestinal inflammation, naive CD4^+^ T cells activated by antigen-presenting cells (APCs) differentiate into subsets including Th1, Th2, Th17, and Treg cells, driving the pathological progression of IBD.^35^ To characterize CD4^+^ T cell polarization in DSS-induced colitis and explore its association with miR-21 expression, we assessed subset-related cytokines, transcription factors, and cellular proportions in colonic tissues and splenic lymphocytes.

In colonic tissues, DSS-treated mice exhibited significant increases in the mRNA levels of the Th1-associated cytokine IFN-γ, the Th2-associated cytokine IL-4, the Th17-associated cytokine IL-17A, and the anti-inflammatory cytokine IL-10, which increased by 3.3-, 2.0-, 47.3-, and 7.5-fold, respectively, compared with the normal water-treated group (Figure 2A). Consistently, enzyme-linked immunosorbent assay (ELISA) confirmed elevated protein levels of IFN-γ, IL-4, IL-17A, and IL-10 in DSS-treated mice (Figure S2). Transcription factor analysis revealed 6.0-fold upregulation of T-bet (Th1), 3.3-fold upregulation of GATA3 (Th2), and 5.0-fold upregulation of RORγt (Th17) but 0.5-fold downregulation of Foxp3 (Treg) mRNA (Figure 2B). Notably, the marked increase in IL-17A and RORγt together with decreased Foxp3 expression suggested a pronounced shift in the Th17/Treg axis during colonic inflammation.

**Figure 2:**
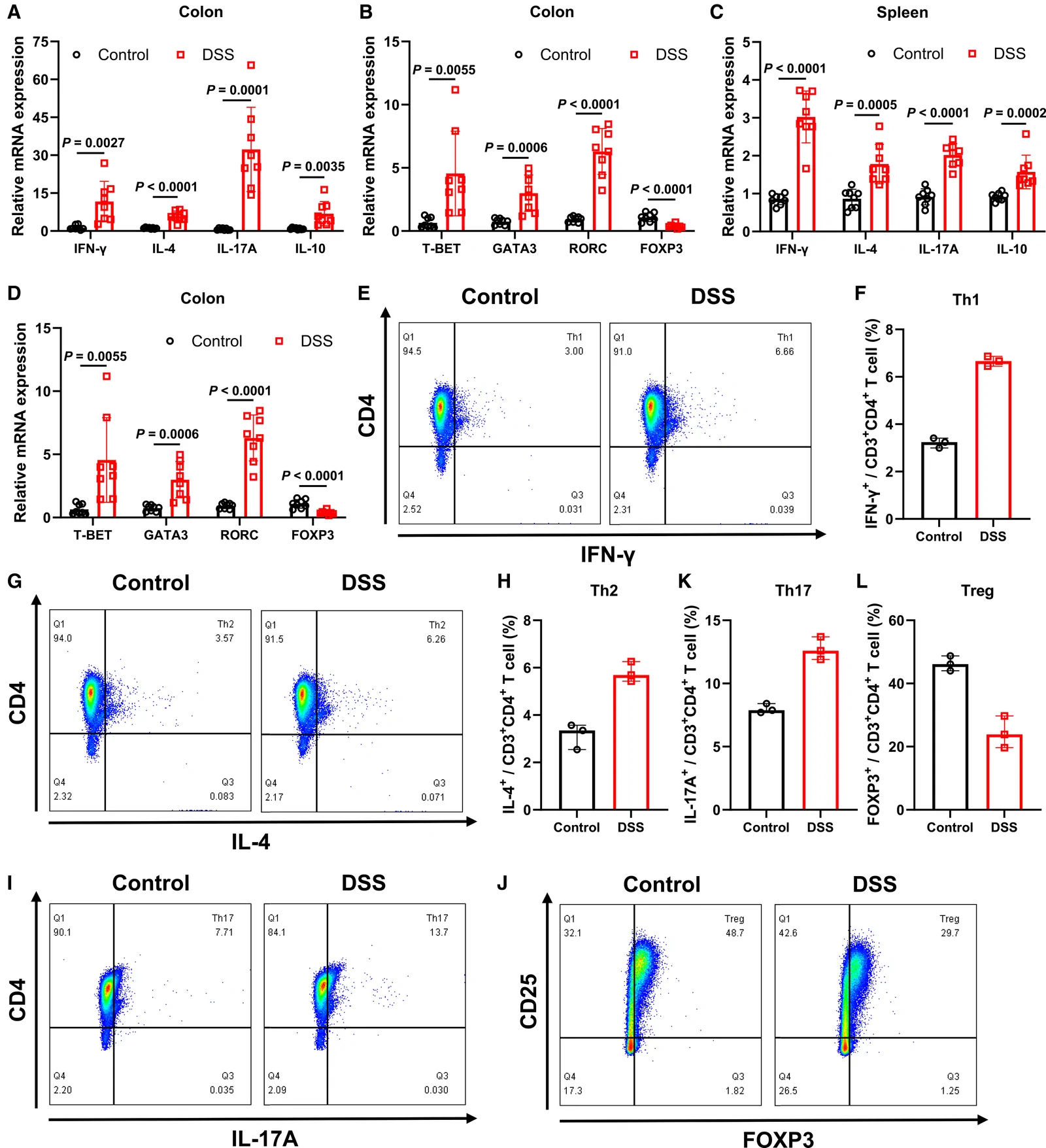
Altered CD4^+^ T cell subset proportions in DSS-induced acute colitis mice (A) RT-qPCR analysis of cytokine mRNA (IFN-γ, IL-4, IL-17A, and IL-10) in mouse colon tissues (*n* = 8). (B) RT-qPCR analysis of transcription factor mRNA (T-bet, GATA3, RORγt, and Foxp3) in mouse colon tissues (*n* = 8). (C) RT-qPCR analysis of cytokine mRNA (IFN-γ, IL-4, IL-17A, and IL-10) in splenic lymphocytes (*n* = 8). (D) RT-qPCR analysis of transcription factor mRNA (T-bet, GATA3, RORγt, and Foxp3) in splenic lymphocytes (*n* = 8). (E) Representative flow cytometry plots for Th1 cell gating. (F) Quantification of Th1 cell frequency among CD3^+^CD4^+^ T cells (*n* = 3). (G) Representative flow cytometry plots for Th2 cell gating. (H) Quantification of Th2 cell frequency among CD3^+^CD4^+^ T cells (*n* = 3). (I) Representative flow cytometry plots for Th17 cell gating. (J) Representative flow cytometry plots for Treg cell gating. (K) Quantification of Th17 cell frequency among CD3^+^CD4^+^ T cells (*n* = 3). (L) Quantification of Treg cell frequency among CD3^+^CD4^+^ T cells (*n* = 3). Data in (A)–(D) were analyzed using the unpaired Student’s *t* test. Data in (F), (H), (K), and (L) were analyzed using the Mann-Whitney U test, and the results are presented as scatterplots with median and 95% confidence interval (CI). Data are presented as the mean ± SD unless otherwise indicated. See also Figure S2.

Interestingly, despite reduced Foxp3 expression, IL-10 levels remained elevated in inflamed colonic tissues. This observation indicates that IL-10 production may not exclusively reflect Treg activity and can be produced by multiple cellular sources, including macrophages, dendritic cells, and type 1 Treg (Tr1) cells.^36^^,^^37^

Similar changes were observed in the spleen. In splenic lymphocytes, DSS treatment similarly increased IFN-γ, IL-4, IL-17A, and IL-10 mRNA levels (Figure 2C); upregulated T-bet, GATA3, and RORγt mRNA levels; and downregulated Foxp3 mRNA levels (Figure 2D). Flow cytometry confirmed elevated proportions of Th1, Th2, and Th17 cells and a reduced Treg frequency among splenic CD3^+^CD4^+^ T cells. Specifically, DSS increased Th1 percentage (median: 6.66% vs. 3.24%, difference: 3.42% [90% confidence interval (CI): 3.04–3.86]), Th2 percentage (median: 5.69% vs. 3.35%, difference: 2.34% [90% CI: 1.86–3.72]), and Th17 percentage (median: 12.60% vs. 7.88%, difference: 4.72% [90% CI: 3.50–5.99]) while decreasing Treg percentage (median: 23.90% vs. 46.10%, difference: −22.2% [90% CI: −29.00 to −14.30]) (Figures 2E–2J). The expansion of Th17 cells together with the reduction of Treg cells mirrored the alterations observed in colonic tissues, indicating that DSS-induced inflammation was associated with both local and systemic disruption of CD4^+^ T cell homeostasis.

Collectively, DSS-induced colitis was characterized by enhanced Th1-, Th2-, and, in particular, Th17-associated responses, accompanied by reduced Treg frequency, indicating a pronounced imbalance in CD4^+^ T cell differentiation, particularly along the Th17/Treg axis. Given the concurrent upregulation of miR-21 in DSS-treated mice, we next investigated whether miR-21 directly contributes to the dysregulation of CD4^+^ T cell differentiation.

### miR-21 promotes Th17 cell differentiation and suppresses Treg cell differentiation

To determine whether miR-21 directly influences CD4^+^ T cell differentiation, we systematically investigated its effects on Th1, Th2, Th17, and induced Treg (iTreg) polarization *in vitro* in combination with functional interventions. Magnetic bead sorting yielded naive CD4^+^ T cells from mouse spleens. These cells were polarized under specific cytokine conditions: Th1 (IL-12 + anti-IL-4), Th2 (IL-4 + anti-IFN-γ), Th17 (IL-6 + TGF-β + anti-IL-4/IFN-γ), and iTreg (IL-2 + TGF-β + anti-IL-4/IFN-γ). Positivity gates were defined using fluorescence-minus-one (FMO) controls as shown in Figure S3. Although the absolute frequencies of the differentiated subsets were relatively modest, the differentiation efficiencies observed in our experiments were consistent with established *in vitro* polarization protocols for naive CD4^+^ T cells (Figures S4A–S4D).^38^^,^^39^^,^^40^ Dynamic monitoring revealed significant upregulation of miR-21 expression during days 4–6 of differentiation across all subsets, with the greatest upregulation observed in Th17-polarized cells (Figure S4E).

Functional validation via transfection with miR-21 mimics or inhibitors demonstrated that miR-21 suppression (inhibitor group) increased Th1 and Treg differentiation frequencies while decreasing Th2 and Th17 proportions vs. inhibitor negative controls (NCs). Conversely, miR-21 overexpression (mimics group) increased Th2 and Th17 differentiation but suppressed Th1 and Treg generation (Figures 3A–3H). These findings demonstrate that miR-21 modulates multiple CD4^+^ T cell differentiation programs, with a particularly pronounced effect on Th17 and Treg differentiation.

**Figure 3:**
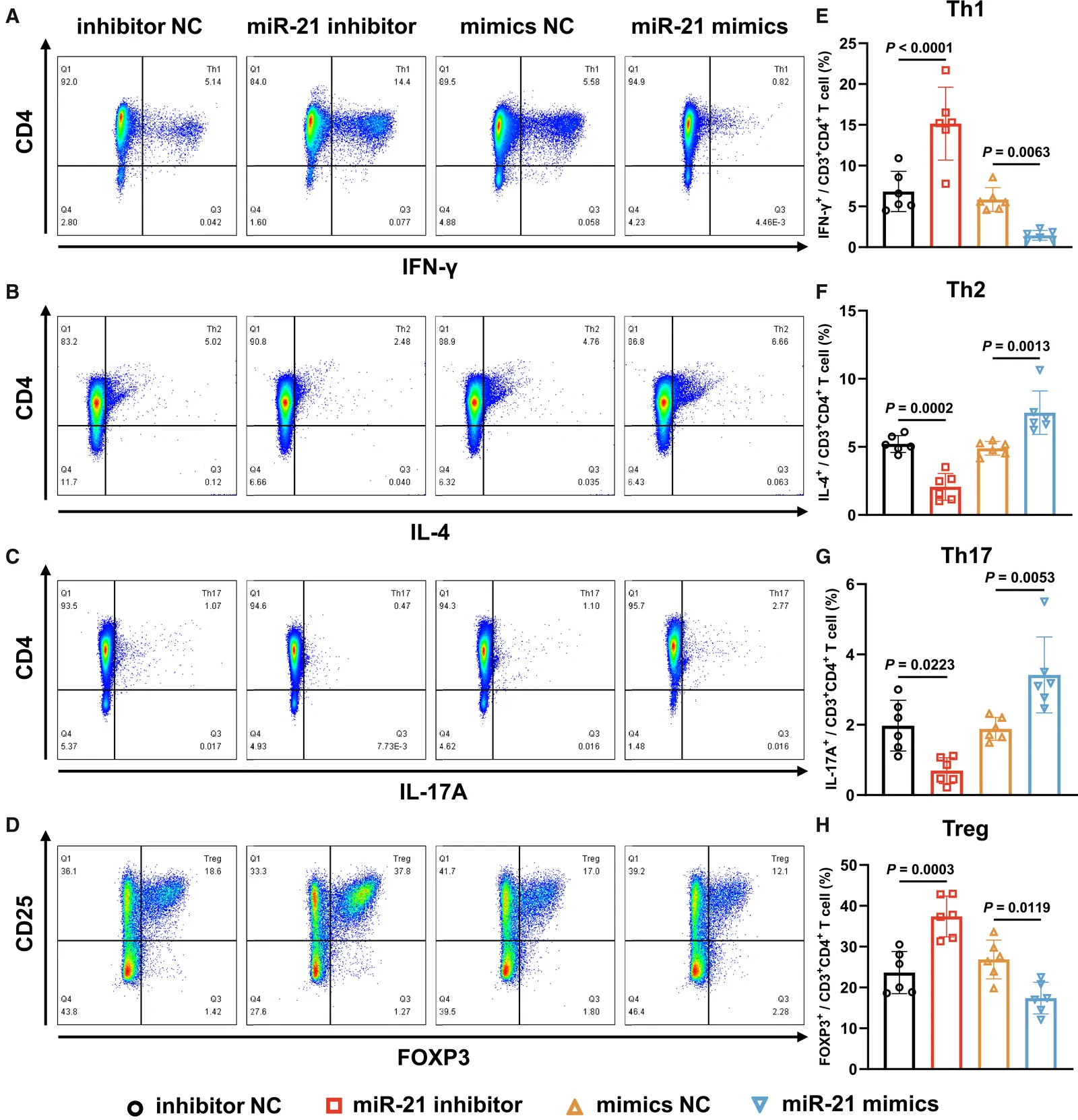
miR-21 promotes Th17 cell differentiation and suppresses Treg cell differentiation (A) Flow cytometry analysis of Th1 cell frequency (CD3^+^CD4^+^IFN-γ^+^) after Th1 polarization. (B) Th2 cell frequency (CD3^+^CD4^+^IL-4^+^) after Th2 polarization. (C) Th17 cell frequency (CD3^+^CD4^+^IL-17A^+^) after Th17 polarization. (D) Treg cell frequency (CD3^+^CD4^+^CD25^+^Foxp3^+^) after Treg polarization. (E–H) Quantification of Th1, Th2, Th17, and Treg cell percentages among CD3^+^CD4^+^ T cells (*n* = 6). Data were analyzed using one-way ANOVA followed by Tukey’s post hoc test. Data are presented as the mean ± SD. See also Figures S3 and S4.

Together with the *in vivo* findings, these results indicate that the increased miR-21 expression observed during DSS-induced colitis may contribute to CD4^+^ T cell dysregulation by promoting Th17 differentiation and suppressing Treg differentiation. Given the prominent involvement of the Th17/Treg axis in the observed immune imbalance, we next investigated the molecular mechanisms underlying miR-21-mediated regulation of Th17 and Treg differentiation.

### Smad7 and Ski are direct targets through which miR-21 regulates Th17/Treg balance

miRNAs exert multi-target regulation by binding to complementary sequences in the 3′ untranslated region (UTR) of target mRNAs via their guide strand, leading to translational repression or degradation.^41^^,^^42^^,^^43^^,^^44^^,^^45^ To decipher the molecular mechanism underlying the miR-21-mediated regulation of Th17/Treg differentiation, we integrated the miRDIP,^46^ miRWalk,^47^ and ENCORI^48^ databases to screen 875 high-probability and 219 extremely high-probability target genes and focused on candidate genes related to the TGF-β pathway and T cell differentiation. Screening identified Smad7 and Ski, both of which are negative regulators of TGF-β signaling, as potential miR-21 targets. Their mRNA expression was significantly reduced in the colons of mice with DSS-induced colitis, concomitant with increased miR-21 expression (Figures 4A–4E). TargetScan prediction and dual-luciferase reporter assays confirmed direct binding of miR-21 to the 3′ UTRs of Smad7 and Ski (Figures 4F and 4G). The miR-21 mimics significantly inhibited wild-type (WT) luciferase activity of Smad7 (median: 0.37 vs. 1.10, difference: −0.73 [90% CI: −0.88 to −0.42]) and Ski (median: 0.70 vs. 1.15, difference: −0.44 [90% CI: −0.66 to −0.25]), whereas the binding activity of the mutant (Mut) was not affected (Smad7 Mut: 1.20 vs. 1.18, difference: 0.02 [90% CI: −0.23 to 0.27]; Ski Mut: 1.03 vs. 1.15, difference: −0.13 [90% CI: −0.31 to 0.06]), indicating Smad7/Ski as the direct target of miR-21.

**Figure 4:**
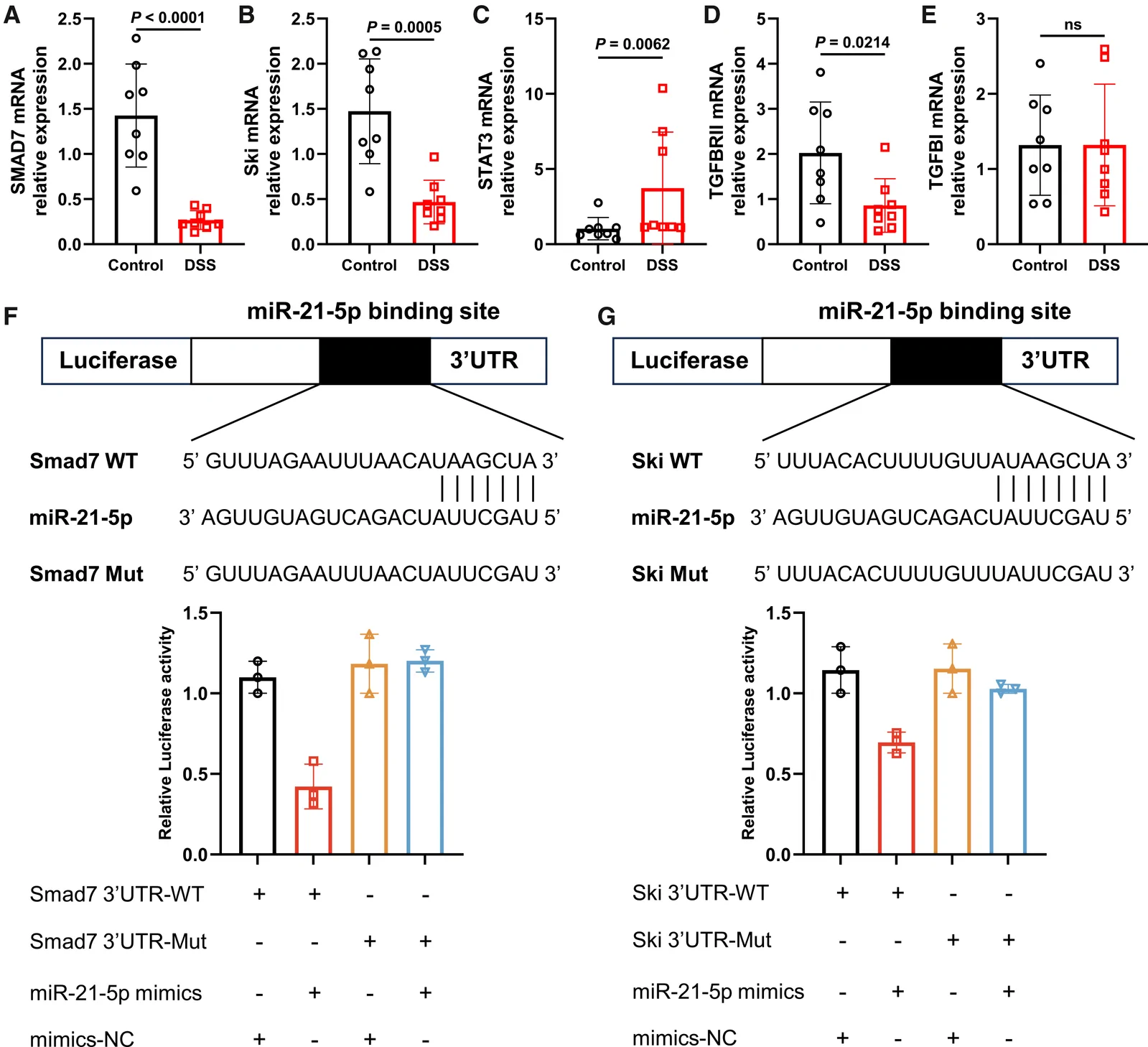
Smad7 and Ski are target genes of miR-21 (A–E) mRNA expression levels of potential miR-21 target genes in the colon tissues of mice with DSS-induced acute colitis, as measured by RT-qPCR (*n* = 8). (F) Predicted binding sites between Smad7 and miR-21, and dual-luciferase reporter assay of Smad7 3′ UTR activity (*n* = 3). (G) Predicted binding sites between Ski and miR-21 and dual-luciferase reporter assay of Ski 3′ UTR activity (*n* = 3). Data in (A)–(E) were analyzed using the unpaired Student’s *t* test. Data in (F) and (G) were analyzed using the Mann-Whitney U test, and the results are presented as scatterplots with median and 90% confidence interval. Data are presented as the mean ± SD unless otherwise indicated.

To determine whether Smad7 and Ski are functionally required for the miR-21-mediated regulation of Th17/Treg balance, we performed rescue experiments in naive CD4^+^ T cells under Th17- or iTreg-polarizing conditions. Cells were co-transfected with miR-21 inhibitor or an inhibitor NC together with si-Smad7, si-Ski, or both, and the frequencies of Th17 and Treg cells were measured by flow cytometry. As shown in Figures 5A–5F, miR-21 inhibitor alone significantly reduced Th17 and increased Treg frequencies, confirming that endogenous miR-21 promotes Th17 and suppresses Treg differentiation. Knockdown of Smad7 or Ski alone mimicked the effect of miR-21 overexpression, leading to increased Th17 and decreased Treg frequencies. Importantly, when the miR-21 inhibitor was co-transfected with si-Smad7 or si-Ski, the effects of the inhibitor were completely reversed, with Th17 and Treg frequencies returning to control levels. Compared with single knockdown, simultaneous knockdown of both Smad7 and Ski did not produce an additive effect. Collectively, these findings support Smad7 and Ski as functionally important downstream mediators of miR-21-mediated regulation of Th17/Treg differentiation and suggest that they may converge on a common regulatory pathway.

**Figure 5:**
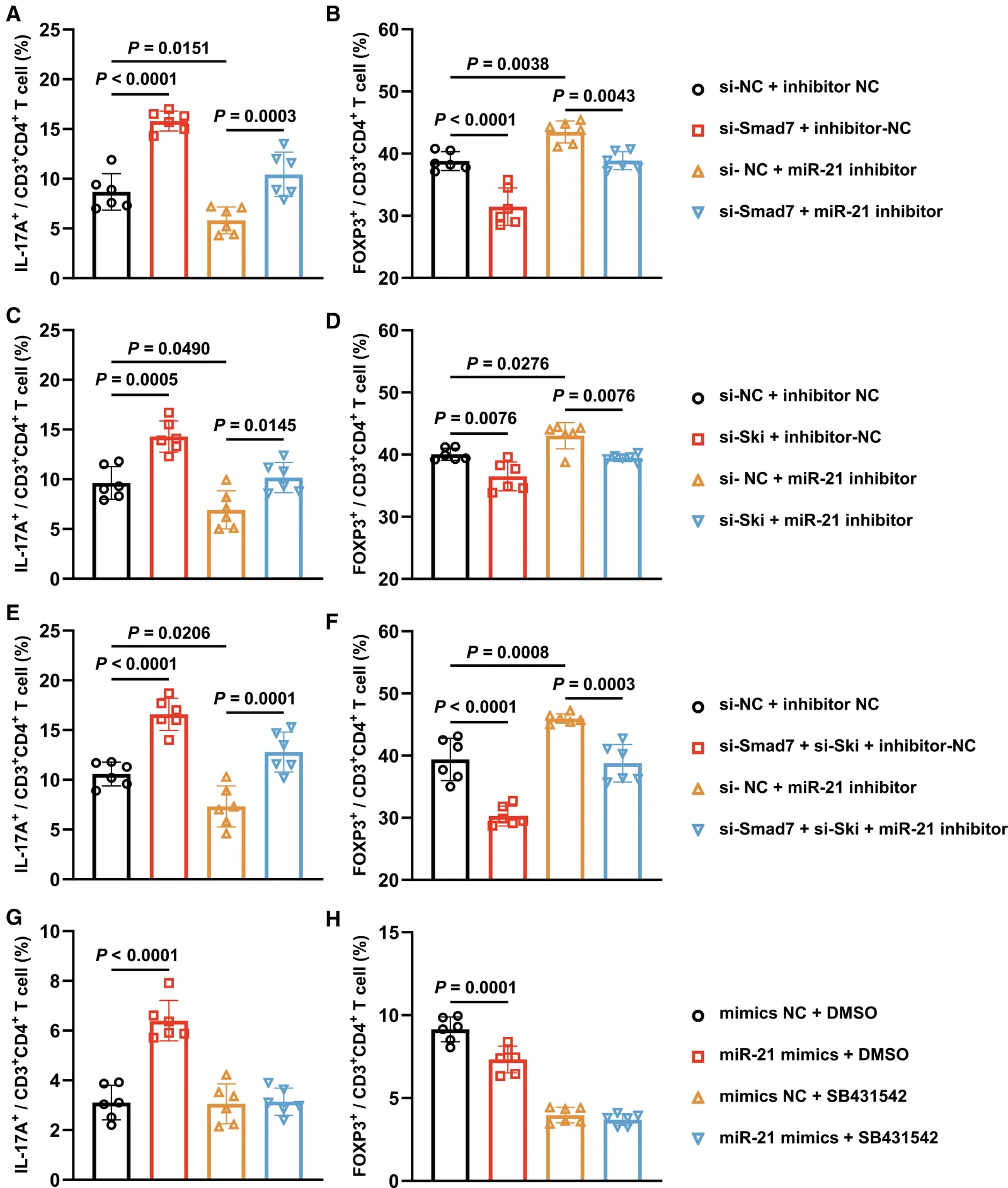
Smad7 and Ski are required for miR-21-mediated regulation of Th17/Treg differentiation, and inhibition of TGF-β signaling abolishes this regulation (A) Quantification of Th17 cell percentages after co-transfection with si-Smad7 and/or miR-21 inhibitor. (B) Quantification of Treg cell percentages under the same conditions as (A). (C) Quantification of Th17 cell percentages after co-transfection with si-Ski and/or miR-21 inhibitor. (D) Quantification of Treg cell percentages under the same conditions as (C). (E) Quantification of Th17 cell percentages after co-transfection with si-Smad7 + si-Ski and/or miR-21 inhibitor. (F) Quantification of Treg cell percentages under the same conditions as (E). (G) Quantification of Th17 cell percentages after treatment with the TGF-β receptor inhibitor SB431542 (10 μM) in the presence or absence of miR-21 mimics. (H) Quantification of Treg cell percentages under the same conditions as (G). Comparisons among multiple groups were performed using one-way ANOVA followed by Tukey’s post hoc test (*n* = 6). Data are presented as the mean ± SD.

To further determine whether TGF-β receptor signaling is involved in miR-21-mediated regulation of Th17/Treg differentiation, we treated differentiating CD4^+^ T cells with the TGF-β receptor inhibitor SB431542. SB431542 largely abolished the effects of miR-21 mimics on both Th17 and Treg differentiation (Figures 5G and 5H). Together with the direct targeting and functional rescue experiments, these findings suggest that miR-21 may modulate TGF-β signaling through coordinated suppression of Smad7 and Ski, thereby contributing to the imbalance between Th17 and Treg differentiation.

### Intervention targeting miR-21 ameliorates DSS-induced acute colitis by regulating Th17/Treg differentiation

To evaluate the therapeutic potential of miR-21 inhibition, we performed therapeutic intervention using a miR-21 antagomir in DSS-induced acute colitis mice. Twenty male C57BL/6 mice received 3% DSS for 3 days to induce acute colitis and were subsequently randomized into four groups: normal control, DSS model, DSS + NC antagomir, and DSS + miR-21 antagomir intervention groups. The latter two groups received intraperitoneal injections of the NC or miR-21 antagomir from day 4 to 9, after which the mice were sacrificed on day 10.

Compared with no treatment, the miR-21 antagomir significantly ameliorated disease phenotypes, as indicated by reduced weight loss (Figure 6A), lower fecal occult blood and DAI scores (Figures 6B and 6C), and attenuated colon shortening (Figures 6D and 6E). Histopathological analysis revealed preserved colonic architecture with minimal inflammatory cell infiltration in the miR-21 antagomir intervention group, accompanied by significantly reduced histological scores (Figures 6F and 6G), indicating substantial mitigation of inflammatory damage. Reverse transcription quantitative polymerase chain reaction (RT-qPCR) confirmed the marked downregulation of miR-21 in colonic tissues and in isolated colonic LPLs of the miR-21 antagomir intervention group (Figures 6H and 6I).

**Figure 6:**
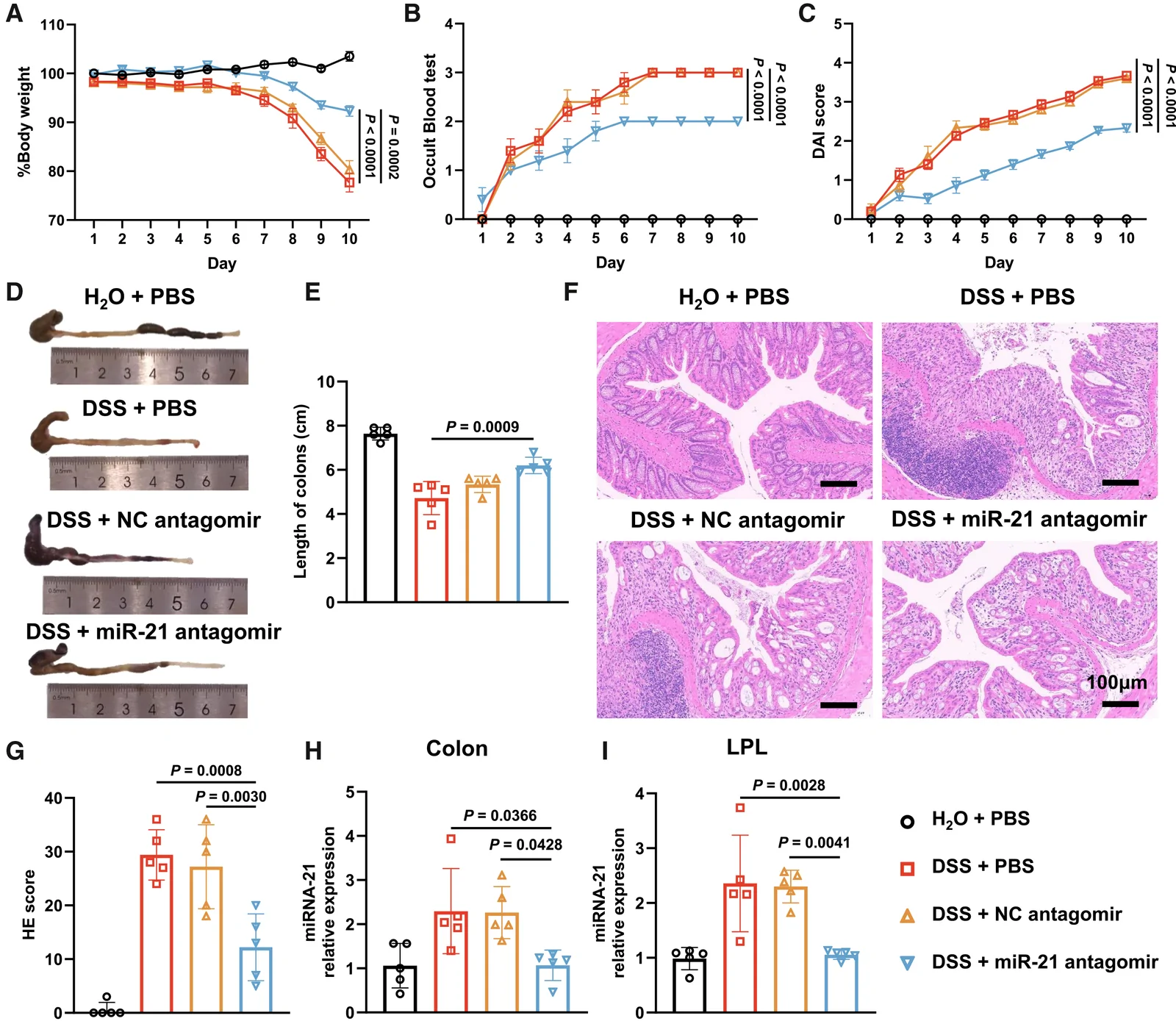
Evaluation of intervention targeting miR-21 (A) Percentage change in body weight (*n* = 5). (B) Fecal occult blood levels (*n* = 5). (C) DAI scores (*n* = 5). (D) Macroscopic appearance of the colons. (E) Quantification of colon length (*n* = 5). (F) Representative H&E-stained images of colon sections (20× magnification). Scale bar: 100 μm. (G) Histopathological scoring of H&E staining (*n* = 5). (H) miR-21 expression levels in colon tissues (*n* = 5). (I) miR-21 expression levels in LPLs (*n* = 5). Data are presented as the mean ± SD. For comparisons among different treatment groups, unpaired Student’s *t* tests with Bonferroni correction for multiple comparisons were performed. Comparisons among multiple groups were analyzed using one-way ANOVA followed by Tukey’s post hoc test. See also Figure S5.

Flow cytometric analysis of colonic LPLs revealed that miR-21 antagomir treatment reduced the frequencies of Th1, Th2, and Th17 cells and increased the frequency of Treg cells compared to DSS + PBS or DSS + NC antagomir groups (Figure 7). qPCR analysis of sorted colonic LPL cells revealed that miR-21 antagomir downregulated the mRNA levels of T-bet, GATA3, and RORγt and the effector cytokines IFN-γ, IL-4, IL-17A, and IL-10 while upregulating Foxp3 expression (Figures S6A and S6B). Correlation analyses further revealed that miR-21 expression was positively correlated with T-bet, GATA3, RORγt, IFN-γ, IL-4, IL-17A, and IL-10 expression and negatively correlated with Foxp3 expression (Figures S6C–S6J). Immunohistochemistry (IHC) confirmed reduced RORγt^+^ cell infiltration in the colonic mucosa (Figures 8A and 8B). Collectively, these findings demonstrate that therapeutic inhibition of miR-21 alleviates DSS-induced acute colitis and is accompanied by restoration of the colonic Th1/Th2/Th17/Treg balance.

**Figure 7:**
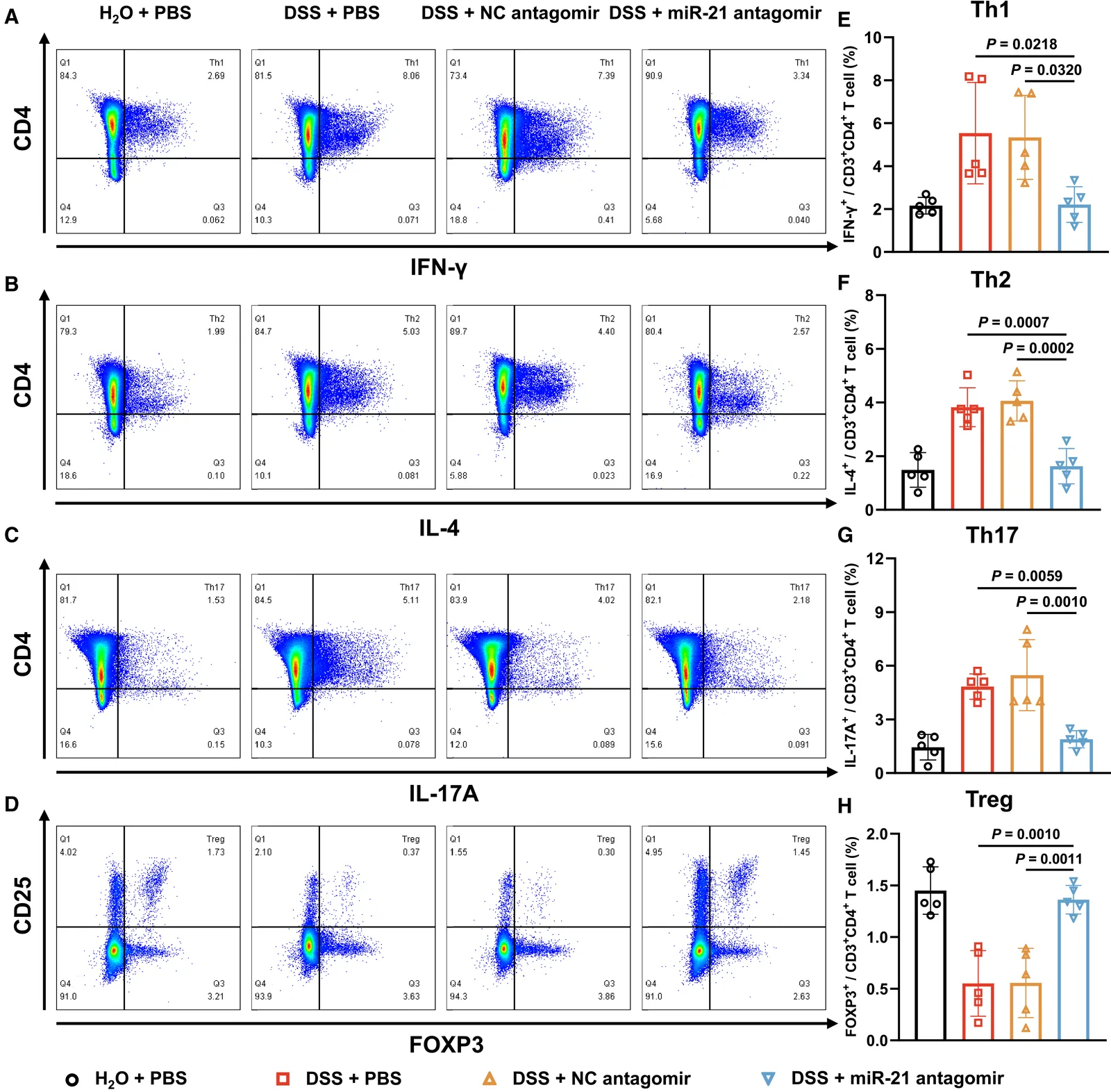
Intervention targeting miR-21 suppresses Th17 cell differentiation and promotes Treg cell differentiation (A–D) Flow cytometry analysis of Th1, Th2, Th17, and Treg cell frequencies in splenic lymphocytes. (E–H) Percentages of Th1, Th2, Th17, and Treg cells among CD3^+^CD4^+^ T cells in splenic lymphocytes (*n* = 5). Comparisons among multiple groups were performed using one-way ANOVA followed by Tukey’s post hoc test. Data are presented as the mean ± SD. See also Figure S6.

**Figure 8:**
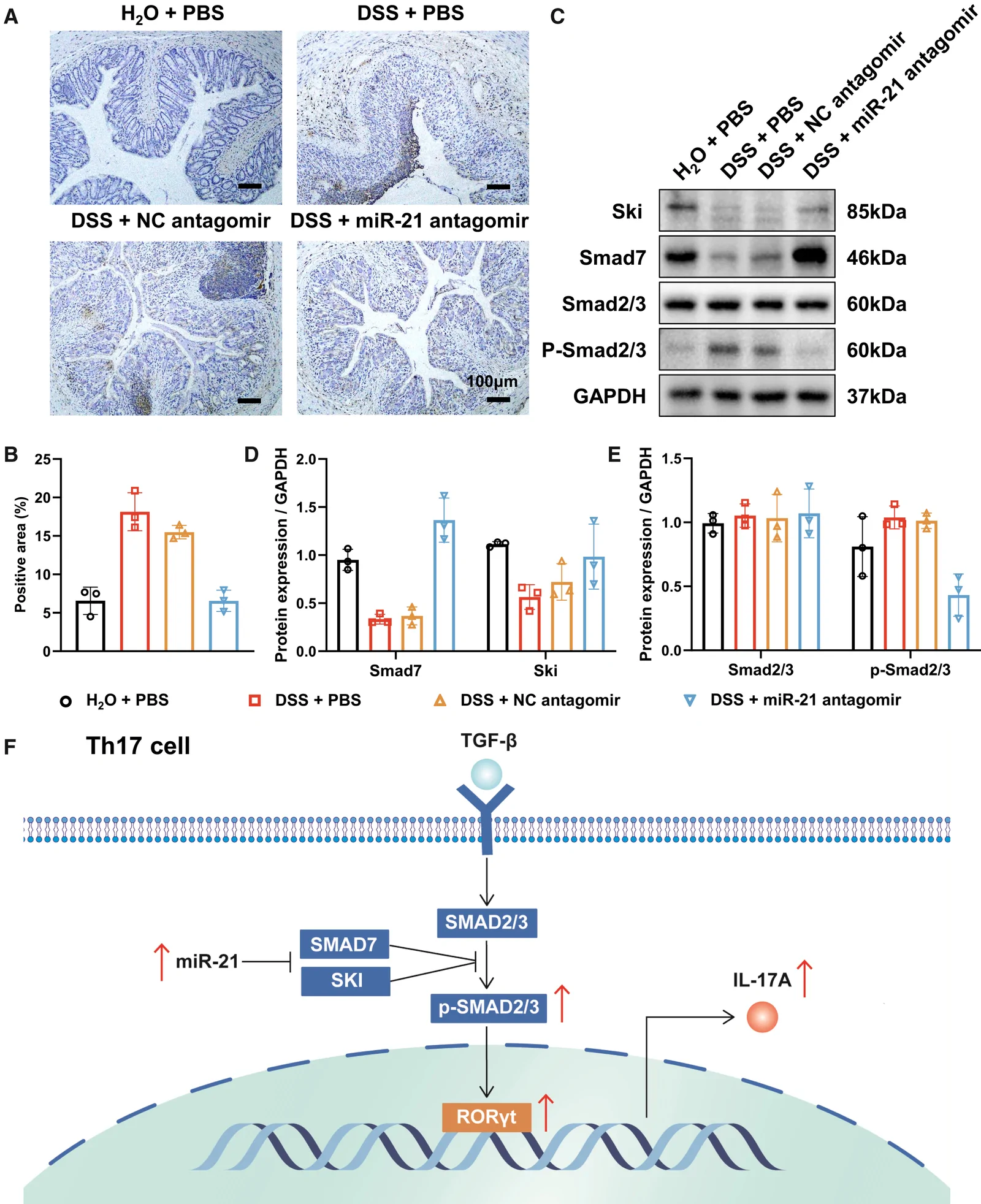
miR-21 activates the p-Smad2/3 signaling pathway by targeting Smad7 and Ski (A) Immunohistochemical staining for RORγt in mouse colon tissues. Positive staining appeared brownish yellow (10× magnification). Scale bar: 100 μm. (B) Quantitative analysis of the relative optical density from IHC staining (*n* = 3). (C) Protein expression of Smad2/3, p-Smad2/3, Smad7, and Ski in protein extracts from colonic lamina propria lymphocytes, as detected by western blot. (D and E) Quantification of Smad2/3, p-Smad2/3, Smad7, and Ski protein levels (*n* = 3). (F) Schematic representation of the proposed miR-21-Smad7/Ski-TGF-β signaling axis involved in Th17 cell differentiation. Data are presented as the mean ± SD. See also Figure S7.

### miR-21 activates p-Smad2/3 signaling by targeting Smad7 and Ski

TGF-β-mediated Smad2/3 phosphorylation is crucial for Th17 differentiation, whereas Smad7 and Ski inhibit Smad2/3-Smad4 complex formation and nuclear translocation through competitive binding to TGF-β receptor I.^49^ This study investigated whether miR-21 influences T cell differentiation by regulating this pathway. To determine whether the miR-21-Smad7/Ski-p-Smad2/3 axis operates in colonic lymphocytes *in vivo*, we performed western blot analysis on protein extracts from colonic LPLs isolated from the indicated experimental groups. As shown in Figures 8C–8E, DSS-induced colitis led to decreased Smad7 and Ski protein levels and increased p-Smad2/3 levels in LPLs. These changes are consistent with enhanced TGF-β signaling and Th17 differentiation and support a model in which miR-21-mediated suppression of Smad7 and Ski relieves negative regulation of TGF-β signaling, thereby contributing to increased Smad2/3 phosphorylation and Th17 differentiation (Figure 8F). Notably, intervention with miR-21 antagomir restored Smad7/Ski protein expression, reduced p-Smad2/3 levels, and was accompanied by a reduction in Th17 differentiation in colon tissues. These results provide *in vivo* evidence supporting the involvement of the miR-21-Smad7/Ski-p-Smad2/3 axis in the regulation of Th17 responses during intestinal inflammation.

To assess the translational relevance of the miR-21-Smad7/Ski-p-Smad2/3 axis, we performed IHC staining for RORγt, Smad7, Ski, and p-Smad2/3 on colonic biopsies from normal controls, patients with active UC, and patients with active CD. Compared with control tissues, active IBD tissues exhibited reduced Smad7 and Ski expression and increased p-Smad2/3 and RORγt^+^ cells (Figure S7). Semi-quantitative analysis confirmed significant differences between the control and active IBD groups. Given the limited sample size, these preliminary human data provide clinical evidence supporting the involvement of the miR-21-Smad7/Ski-p-Smad2/3 axis in IBD.

## Discussion

In the present study, we investigated the role of miR-21 in intestinal inflammation and its impact on CD4^+^ T cell differentiation in IBD. Consistent with previous reports,^17^^,^^50^ miR-21 expression was significantly elevated in inflamed intestinal tissues from patients with IBD and in DSS-induced colitis and was associated with enhanced inflammatory responses and disruption of CD4^+^ T cell homeostasis. Importantly, rather than establishing a previously unrecognized role for miR-21 in T cell differentiation, our findings extend existing knowledge by identifying Ski as an additional functional target through which miR-21 regulates TGF-β signaling. We demonstrate that simultaneous suppression of Smad7 and Ski by miR-21 enhances Smad2/3 activation, promotes Th17/Treg imbalance, and exacerbates intestinal inflammation. Furthermore, pharmacological inhibition of miR-21 restored Smad7 and Ski expression, reduced Smad2/3 activation, and ameliorated experimental colitis. Together, these findings support the existence of a broader miR-21-Smad7/Ski-TGF-β regulatory network during intestinal inflammation.

Accumulating evidence has implicated miR-21 in the pathogenesis of IBD.^14^^,^^17^^,^^50^ Increased miR-21 expression has been reported in intestinal mucosa, peripheral blood, and immune cells from patients with active disease, suggesting a potential role in both disease progression and immune regulation. Consistent with these observations, we detected significant upregulation of miR-21 in inflamed tissues from patients with UC and CD as well as in DSS-treated mice. Moreover, miR-21 expression positively correlated with inflammatory severity. Thorlacius-Ussing et al. reported that miR-21 is localized in CD68^+^ macrophages and CD3^+^ T cells in the colonic mucosa of patients with UC.^50^ This localization suggests that miR-21 may participate in the pathological process of colitis through effects on multiple immune cell populations, including T cells. Although most studies support a pathogenic role for miR-21 in IBD, discrepant findings have occasionally been reported.^21^^,^^22^ Such variability may reflect differences in sample source, disease stage, treatment exposure, cellular composition, or analytical methodologies. The concordant findings observed in both clinical specimens and experimental colitis in the present study strengthen the evidence supporting miR-21 as a contributor to active intestinal inflammation.

Dysregulated CD4^+^ T cell responses represent a central feature of IBD pathogenesis. In agreement with previous studies,^51^^,^^52^^,^^53^^,^^54^ DSS-induced colitis was associated with increased Th1, Th2, and Th17 responses together with reduced Treg-associated signatures. Among these alterations, the most prominent changes involved the Th17/Treg axis, characterized by marked induction of IL-17A and RORγt and suppression of Foxp3 expression.^8^^,^^54^ Notably, the observed reduction in Tregs in our model diverges from the findings of several clinical studies reporting increased Tregs in inflamed tissues,^55^^,^^56^ potentially attributable to distinct pathological features of the acute DSS-induced colitis model compared with those of chronic human IBD.^57^^,^^58^ Although Th17/Treg imbalance has been widely implicated in chronic intestinal inflammation, the upstream molecular regulators governing this process remain incompletely understood. Our findings suggest that elevated miR-21 contributes to this imbalance by favoring differentiation toward proinflammatory effector subsets while impairing Treg development.

Previous studies have already established an important role for miR-21 in regulating CD4^+^ T cell differentiation in multiple immune-mediated diseases. In particular, miR-21 has been implicated in promoting Th17 differentiation and regulating the balance between Th17 and Treg cells through context-dependent effects on TGF-β signaling and other regulatory pathways.^29^^,^^30^^,^^33^^,^^59^ miR-21 deficiency has been shown to alleviate disease in models of EAE, multiple myeloma, and chronic graft-vs.-host disease, which is consistent with our findings.^29^^,^^30^^,^^59^ Moreover, Rossi et al. found that inhibition of miR-21 may lead to the redirection of Th17 cells toward Th1/Th2-like activated or polarized phenotypes, suggesting that miR-21 may also participate in the regulation of Th17 plasticity.^30^ Interestingly, the effects of miR-21 inhibition on Th1 responses differed between our *in vitro* and *in vivo* models. *In vitro*, miR-21 inhibition increased Th1 differentiation. However, whether this increase reflects plasticity-driven conversion of Th17 cells toward a Th1 phenotype remains unknown. *In vivo*, therapeutic administration of a miR-21 antagomir reduced the frequency of colonic Th1 cells in DSS-induced colitis, which could theoretically contribute to disease attenuation. Notably, the reported relationship between miR-21 and Th1 responses has also yielded conflicting findings across different experimental settings.^29^^,^^30^^,^^60^ This discrepancy may reflect differences between T cell-intrinsic responses under controlled polarization conditions and the complex inflammatory microenvironment *in vivo*. Consistent with the findings of Murugaiyan et al., which suggested that miR-21 is not essential for Th1 differentiation but plays a more prominent role in Th17 differentiation, our study primarily focused on the role of miR-21 in Th17 differentiation during intestinal inflammation.^29^ Taken together, our findings and previous studies support a role for miR-21 in regulating Th17 responses, with the specific downstream consequences likely depending on the disease context.

These findings prompted us to further explore the molecular mechanisms underlying the effects of miR-21 on Th17/Treg differentiation. A major finding of the present study is the identification of Ski as a direct and functionally relevant target of miR-21 in the regulation of Th17/Treg differentiation. Previous mechanistic studies have primarily focused on Smad7 as the critical mediator linking miR-21 to TGF-β signaling.^29^ Consistent with these reports,^29^ we confirmed that miR-21 directly suppresses Smad7 expression and enhances downstream Smad2/3 activation. Importantly, we further demonstrated that Ski is directly targeted by miR-21 and functionally contributes to the effects of miR-21 on CD4^+^ T cell differentiation. These findings substantially expand the previously described miR-21-Smad7 regulatory model by identifying Ski as an additional functional component of this regulatory network.

The biological significance of Ski is particularly noteworthy because Ski and Smad7 regulate TGF-β signaling through distinct yet complementary mechanisms. Smad7 functions primarily as an inhibitory Smad that blocks receptor-mediated phosphorylation of Smad2/3, whereas Ski acts as a transcriptional co-repressor that suppresses TGF-β-responsive gene transcription through interactions with Smad complexes and chromatin-modifying proteins.^61^^,^^62^ Consequently, these molecules exert inhibitory effects at different levels of the signaling cascade. Importantly, because both Smad7 and Ski are intracellular negative regulators of TGF-β signaling, their effects should be interpreted in terms of TGF-β signaling intensity rather than changes in extracellular TGF-β concentration.^63^^,^^64^ Our data suggest that simultaneous repression of Smad7 and Ski by miR-21 amplifies TGF-β signaling more effectively than inhibition of either target alone, thereby promoting Th17 differentiation and suppressing Treg generation. These findings support a model in which miR-21 coordinates multiple inhibitory checkpoints within the TGF-β pathway to modulate the balance between Th17 and Treg differentiation.

Interestingly, the identification of Ski may also provide a potential framework for understanding the heterogeneous patterns of Smad7 expression reported in previous IBD studies.^65^^,^^66^ Monteleone reported Smad7 overexpression in lamina propria T cells from inflamed IBD mucosa,^65^ whereas Judith Abarca-Zabalía reported downregulation of Smad7 in peripheral blood CD4^+^ T cells from patients with CD.^66^ Other studies have similarly described differences in Smad7 expression according to tissue, cell population, and disease context.^67^^,^^68^ The low expression state of Smad7/Ski observed in our acute inflammatory model is similar to the low-Smad7-expression phenotype described by Salvador-Martín in anti-tumor necrosis factor (TNF) non-responders^67^ and in lamina propria T cells from patients with IBD and colorectal cancer.^68^ Although our study was not designed to resolve these discrepancies, the simultaneous regulation of multiple inhibitory nodes within the TGF-β pathway by miR-21 may contribute to the context-dependent regulation of TGF-β signaling. Longitudinal and cell-specific studies will be required to determine whether the relative contribution of these regulatory mechanisms changes across different stages of IBD.

From a translational perspective, intraperitoneal administration of miR-21 antagomir produced substantial therapeutic benefits in DSS-induced colitis. Administration of miR-21 antagomir reduced disease severity, restored expression of both Smad7 and Ski, suppressed Smad2/3 activation, and was accompanied by restoration of the Th17/Treg imbalance. These results provide a mechanistic complement to Johnston’s observation of increased resistance to DSS-induced acute colitis in miR-21 knockout mice.^69^ Nevertheless, the pleiotropic functions of miR-21 across multiple tissues necessitate caution regarding systemic inhibition.^70^ Although chemically modified antagomirs and targeted delivery systems hold promise for enhancing therapeutic specificity,^71^^,^^72^^,^^73^ their clinical translation still faces challenges such as hepatotoxicity and insufficient cellular targeting. Future development of gut-targeted delivery systems and cell-specific oligonucleotide strategies may improve therapeutic specificity and facilitate clinical translation.

This study has several limitations. First, the limited clinical sample size restricted the validation of intestinal CD4^+^ T cell subsets and Smad7/Ski-p-Smad pathway proteins in patients with IBD. Second, the hierarchical interaction between Smad7 and Ski within the regulatory network remains unclear. Third, while we established the direct binding and functional necessity of Smad7 and Ski through dual-luciferase and small interfering RNA (siRNA) rescue experiments, we did not perform overexpression rescue experiments with 3′ UTR-mutated constructs due to technical difficulties in primary T cell transfection. This remains a goal for future studies to further confirm target specificity. Fourth, mechanistic studies were conducted primarily in splenic naive CD4^+^ T cells rather than intestinal lamina propria T cells. Although this system provides a controlled approach for assessing T cell-intrinsic effects, it does not fully recapitulate the complex intestinal inflammatory microenvironment, which may contribute to the differences observed between our *in vitro* and *in vivo* findings, particularly for Th1 responses. Fifth, the acute DSS model better recapitulates UC than CD. Subsequent experiments should be replicated in chronic colitis models and CD-like models (e.g., T cell transfer models). Finally, the upstream regulatory mechanisms of miR-21 and the basis for its tissue-specific expression require further exploration. Future research should investigate these mechanisms and determine how the relative contributions of Smad7 and Ski change across different stages and cellular compartments of IBD.

In summary, this study expands the current understanding of miR-21 biology in intestinal inflammation by identifying Ski as an additional functional target alongside Smad7. Coordinated suppression of Smad7 and Ski enhances TGF-β/Smad2/3 signaling and contributes to Th17/Treg dysregulation in experimental colitis. These findings provide a broader framework for understanding the multi-target effects of miR-21 in intestinal inflammation and support further investigation of miR-21-targeted strategies for UC.

## Materials and methods

### Experimental animals and human tissue specimens

Specific pathogen-free (SPF) male C57BL/6 mice (6–8 weeks old, uniform body weight) were supplied by Hubei Biont Biological Technology. All animals were housed in the SPF barrier facility at the Experimental Animal Center of Tongji Hospital (facility license no. SYXK (Hubei) 2019-0106) under the following controlled conditions: temperature 24.0°C ± 2.0°C, humidity 65.0% ± 10%, and a 12 h/12 h light/dark cycle. All animal procedures were approved by the Animal Welfare Ethics Committee of Tongji Hospital (approval no. TJH-202209003).

Human intestinal mucosal tissue specimens were obtained from patients diagnosed with IBD and intestinal polyps at Tongji Hospital, Tongji Medical College, Huazhong University of Science and Technology. The collection of human specimens was approved by the Medical Ethics Committee of Tongji Hospital (approval no. TJ-IRB20230404). Patient enrollment strictly followed the diagnostic criteria outlined in the Chinese Consensus on the Diagnosis and Treatment of Inflammatory Bowel Disease.^74^ Individuals with severe cardiopulmonary diseases or other autoimmune disorders were excluded. Disease activity was assessed using the best Crohn’s DAI (CDAI) for CD and the modified Mayo score for UC. Endoscopic activity was graded according to the Chinese Grading System of Crohn 's Disease (CGSCD) endoscopy score for CD and the Mayo endoscopy score for UC.

### DSS-induced acute colitis mouse model

After a 7-day acclimatization period, the mice were administered freshly prepared 3% (w/v) DSS (MP Biomedicals) solution *ad libitum* (approximately 8 mL per mouse per day, replaced every 48 h). At a fixed time daily, the following parameters were recorded: (1) body weight change (scored 0–4), (2) stool consistency (scored 0–4: normal to diarrhea), and (3) fecal occult blood (scored 0–4: assessed by color development time/intensity using an orthotolidine-based kit from Nanjing Jiancheng Bioengineering Institute, Nanjing, China. The DAI was calculated as the sum of these three scores divided by 3.

On day 7, the mice were euthanized via cervical dislocation following blood collection from the orbital sinus. Spleens were aseptically excised and placed in ice-cold phosphate buffered saline (PBS). The entire colon was resected, its length was measured, and the colon was then processed as follows: (1) a 1-cm segment of the distal colon was fixed in *in situ* hybridization fixative (Servicebio, Wuhan, China) and (2) the remaining tissue was aliquoted and snap-frozen for subsequent RNA extraction, protein extraction, MPO activity detection, and ELISA.

### MPO activity assay

Colon tissue was weighed (wet weight) and homogenized on ice in Reagent 2 (Nanjing Jiancheng Bioengineering Institute kit, Nanjing, China) at a 1:19 (w/v) ratio to prepare a 5% tissue homogenate. This homogenate was then mixed with Reagent 3 at a 9:1 ratio and incubated at 37°C for 15 min. The reaction system was established according to the manufacturer’s instructions. After incubation in a 37°C water bath for 30 min, 0.05 mL of Reagent 7 was added, followed by a further 10-min incubation in a 60°C water bath. The absorbance was measured at 460 nm. MPO activity (U/g tissue) was calculated using the following formula: MPO activity = (A_sample − A_control)/(11.3 × tissue wet weight (g)).

### Paraffin sectioning and H&E staining

Colon tissues were fixed in 4% paraformaldehyde for 24–48 h, dehydrated through a graded ethanol series, cleared in xylene, and embedded in paraffin. Serial sections of 4 μm thickness were prepared using a microtome, baked at 60°C until anhydrous, and then subjected to the following staining procedure: deparaffinization and rehydration, staining with hematoxylin for 5 min, treatment with a differentiation solution, neutralization with a bluing reagent, counterstaining with eosin for 5 min, dehydration through a graded ethanol series, and mounting with neutral resin.

Histopathological evaluation was performed using an OLYMPUS light microscope. The following parameters were assessed and scored: (1) inflammation severity (0–3: none → transmural infiltration), (2) damage depth (0–3: none → full thickness), and (3) crypt destruction (0–4: intact → complete loss). The final score is the sum of the sub-item scores multiplied by the lesion area weight coefficient.

### FISH

Paraffin-embedded sections were deparaffinized and rehydrated. The sections were subsequently treated with Proteinase K working solution (Genepharma, Shanghai, China) and digested at 37°C for 20 min. Afterward, the sections were incubated in denaturation solution at 78°C for 8 min. A 1 μM mmu-miR-21-5p fluorescent probe (Genepharma) hybridization solution was applied to the sections, which were then coverslipped and incubated in a humidified chamber at 37°C for 16 h. After hybridization, the sections were washed in washing buffer at 43°C for 15 min, counterstained with DAPI (Servicebio) for 10 min, and finally mounted with an anti-fade mounting medium (Servicebio). Images were acquired using an OLYMPUS fluorescence microscope.

### Quantitative real-time PCR

Total RNA was extracted from tissues or cells using RNAiso Plus reagent (Takara, Beijing, China). RNA concentration and purity were determined using a NanoDrop 2000 spectrophotometer (Thermo Fisher Scientific). Reverse transcription was performed using a PrimeScript RT reagent Kit (Takara) according to the manufacturer’s instructions. In brief, 1 μg of total RNA was reverse transcribed in a 10 μL reaction volume containing Oligo dT primers, Random 6 mers, and a specific stem-loop RT primer for miR-21, under the following conditions: 42°C for 15 min, followed by 85°C for 5 s.

The resulting cDNA was diluted 10-fold and subjected to qPCR amplification using TB Green Premix Ex Taq II (Takara) on a 7900HT Fast Real-Time PCR System (Thermo Fisher Scientific). The thermal cycling protocol was as follows: initial denaturation at 95°C for 30 s and 40 cycles of 95°C for 5 s and 60°C for 30 s. All primer sequences are listed in Table S1 (synthesized by Wuhan Aoke Dingsheng Biotechnology, Wuhan, China). U6 small nuclear RNA and GAPDH mRNA were used as endogenous controls for miRNA and mRNA normalization, respectively. The relative expression levels were calculated using the comparative 2^−ΔΔCT^ method.

### Western blot

Colonic LPLs were isolated as described above, washed twice with ice-cold PBS, and lysed on ice for 30 min using radio immunoprecipitation assay (RIPA) lysis buffer (Servicebio) supplemented with a protease and phosphatase inhibitor cocktail (New Cell & Molecular Biotech, Suzhou, China). The lysates were then centrifuged at 12,000 rpm for 10 min at 4°C, after which the supernatant was collected. The protein concentration was determined using a bicinchoninic acid (BCA) protein assay kit (Servicebio). The proteins were subsequently denatured by boiling with 5× sodium dodecyl sulfate (SDS) loading buffer (Servicebio) for 10 min.

Equal amounts of protein were separated by 10% SDS-polyacrylamide gel electrophoresis (PAGE) electrophoresis and then transferred onto a polyvinylidene fluoride (PVDF) membrane (Millipore) using a wet transfer system. The membrane was blocked with 5% bovine serum albumin (BSA) in Tris-buffered saline with tween-20 (TBST) for 2 h at room temperature, followed by incubation with specific primary antibodies diluted in blocking buffer overnight at 4°C. The primary antibodies used were as follows: PDCD4 Rabbit pAb, Smad7 Rabbit pAb, SKI Rabbit pAb, Smad2/Smad3 Rabbit pAb, Phospho-Smad2 (Ser465/467)/Smad3 (Ser423/425) Rabbit pAb, Anti-beta Actin Mouse mAb, and Anti-GAPDH Mouse mAb (all from ABclonal). After extensive washing, the membrane was incubated with corresponding horseradish peroxidase (HRP)-conjugated secondary antibodies (Goat Anti-Rabbit or Goat Anti-Mouse IgG, Servicebio) for 2 h at room temperature.

The protein bands were visualized using a supersensitive enhanced chemiluminescence (ECL) substrate (New Cell & Molecular Biotech), and images were captured with a Tanon imaging system. Densitometric analysis was performed using ImageJ software.

### Naive CD4^+^ T cell isolation and *in vitro* polarization

Mouse spleens were gently homogenized by grinding through a 200G mesh screen. Mononuclear cells were isolated from single-cell suspensions using mouse spleen lymphocyte separation medium (Tianjin Haoyang, Tianjin, China) via density gradient centrifugation. Approximately 2 × 10^7^ mononuclear cells were typically obtained per mouse spleen.

Naive CD4^+^ T cells were subsequently purified from the mononuclear cell fraction using a Mouse Naive CD4^+^ T cell Isolation Kit (Univ-bio, Shanghai, China) by negative selection for CD44^+^ cells. Briefly, the cells were incubated with a biotin-antibody cocktail, followed by anti-biotin microBeads and CD44 microBeads at 4°C. Labeled cells were then depleted using LS separation columns (Univ-bio) according to the manufacturer’s protocol. The typical yield was approximately (3–6) × 10^6^ naive CD4^+^ T cells per mouse spleen.

The isolated naive CD4^+^ T cells were resuspended in T cell polarization medium (RPMI-1640 supplemented with 10% fetal bovine serum (FBS) and 1% penicillin-streptomycin) at a density of (0.5–1) × 10^6^ cells/mL and seeded into 24-well plates precoated with anti-mouse CD3 antibody (10 μg/mL, BioLegend). The cells were polarized under the following cytokine and antibody conditions for 72 h: Th1, IL-12 (15 ng/mL, PeproTech) + anti-IL-4 (5 μg/mL, BioLegend) + anti-CD28 (2 μg/mL, BioLegend); Th2, IL-4 (30 ng/mL, PeproTech) + anti-IFN-γ (5 μg/mL, BioLegend) + anti-CD28 (2 μg/mL); Th17, IL-6 (20 ng/mL, PeproTech) + TGF-β (4.5 ng/mL, PeproTech) + anti-IL-4 (5 μg/mL) + anti-IFN-γ (5 μg/mL) + anti-CD28 (2 μg/mL); and iTreg, IL-2 (10 ng/mL, PeproTech) + TGF-β (15 ng/mL) + anti-IL-4 (5 μg/mL) + anti-IFN-γ (5 μg/mL) + anti-CD28 (2 μg/mL). After the 72-h polarization period, cells and culture supernatants were collected for subsequent analysis.

### Cell transfection

Isolated naive CD4^+^ T cells were seeded into 24-well plates at a density of 2 × 10^5^ cells per well 1 day prior to transfection. Transfection was performed using HiPerFect Transfection Reagent (Qiagen) according to the manufacturer’s instructions for the following reagents: (1) miR-21 inhibitor, miR-21 mimics, or their respective NCs (GenePharma, Shanghai, Wuhan, final concentration: 50 nM) and (2) Smad7/Ski siRNA or NC siRNA (GenePharma, final concentration: 100 nM). Six hours after transfection, the medium was replaced with fresh polarization medium. After 72 h of culture, the transfection was considered complete, and cells were ready for subsequent experiments.

### Flow cytometry

The cells were prestimulated for 5 h using a Leukocyte Activation Cocktail (with BD GolgiPlug, Univ-bio). The cells were subsequently stained with Fixable Viability Stain 510 (Univ-bio) for 15 min in the dark, followed by washing with PBS.

Surface staining was performed by incubating the cells with the following antibodies for 30 min at 4°C in the dark: anti-mouse CD3e-FITC (clone 145-2C11, Univ-bio) and anti-mouse CD4-BB700 (clone RM4-5, Univ-bio).

For intracellular cytokine staining, the cells were fixed and permeabilized using the Cytofix/Cytoperm Solution Kit (Univ-bio) according to the manufacturer’s instructions. The cells were then stained intracellularly with the following antibodies for 30 min at 4°C in the dark: anti-mouse IFN-γ-PE (clone XMG1.2; Univ-bio), anti-mouse IL-4-PE (clone 11B11; Univ-bio), and anti-mouse IL-17A-Alexa Fluor 647 (clone TC11-18H10; Univ-bio).

For transcription factor staining (Foxp3), cells were fixed and permeabilized using the Transcription Factor Buffer Set (Univ-bio) according to the manufacturer’s protocol. Nuclear staining was performed using anti-mouse Foxp3-PE (clone MF-23; Univ-bio) for 60 min at 4°C in the dark.

Data acquisition was performed on a Beckman CytoFLEX flow cytometer. The frequencies of Th cell subsets were analyzed using FlowJo software (v.10) and defined as follows: Th1, CD3^+^CD4^+^IFN-γ^+^; Th2, CD3^+^CD4^+^IL-4^+^; Th17, CD3^+^CD4^+^IL-17A^+^; and Treg, CD3^+^CD4^+^CD25^+^Foxp3^+^.

### ELISA

Colon tissue was homogenized on ice in normal saline at a 1:9 (w/v) ratio. The homogenate was then centrifuged at 4,000 rpm for 10 min at 4°C, after which the supernatant was collected for analysis. The cell culture supernatants were assayed directly.

The assay was performed using commercial ELISA kits (Boster, Wuhan, China) according to the manufacturer’s instructions. Briefly, 100 μL of each sample or standard was coated onto a plate. The plates were subsequently incubated with biotinylated detection antibody working solution for 1 h at 37°C, followed by incubation with avidin-biotin-peroxidase complex (ABC) working solution for 30 min at 37°C. Color development was initiated by adding 3,3′,5,5′-tetramethylbenzidine (TMB) substrate solution and incubating for 25 min at 37°C. The reaction was stopped by adding stop solution, and the absorbance was immediately measured at 450 nm using a Thermo Fisher Scientific microplate reader. The concentrations of IFN-γ, IL-4, IL-17A, and IL-10 were interpolated from their respective standard curves and are expressed in pg/mL.

### Dual-luciferase reporter assay

Dual-luciferase reporter vectors containing the WT or Mut 3′ UTR sequences of Smad7 or Ski were constructed by Genepharma. HEK293T cells were seeded into 24-well plates at a density of 5 × 10^4^ cells per well. After 24 h, the cells were co-transfected using HiPerFect Transfection Reagent (Univ-bio) with the following: (1) miR-21 mimics, miR-21 inhibitor, or their respective NCs (at a final concentration of 50 nM) and (2) either the WT or Mut dual-luciferase reporter vector (at a final concentration of 10 nM). Forty-eight hours after transfection, the cells were lysed. Firefly and Renilla luciferase activities were measured sequentially using a Dual-Luciferase Reporter Assay System (Yeasen, Shanghai, China) according to the manufacturer’s protocol.

### Intervention experiment

To evaluate the therapeutic potential of miR-21 inhibition, mice were given 3% DSS in their drinking water for 3 days to induce acute colitis. On day 4, the mice were randomized into four groups (*n* = 5 per group): (1) H_2_O + PBS, (2) DSS + PBS, (3) DSS + NC antagomir, and (4) DSS + miR-21 antagomir. Antagomir (5 mg/kg, Genepharma) or an equal volume of PBS was injected intraperitoneally every other day from day 4 to 9. The mice were euthanized on day 10 for sample collection. The DAI was monitored daily.

### Isolation of colonic LPLs

Colons were harvested, cleared of contents and mucus, and opened longitudinally. To remove epithelial cells and intraepithelial lymphocytes, tissues were washed three times with D-Hank's balanced salt solution (DHBSS) and incubated in ethylenediaminetetraacetic acid (EDTA)-DL-dithiothreitol (DTT) working solution (5 mM EDTA, 1 mM DTT, and 1% FBS in DHBSS) for 5 min at 37°C with shaking, followed by vortexing for 10 s. After three washes in RPMI-1640, tissues were cut into 0.2–0.5 cm pieces and digested in complete medium supplemented with 1 mg/mL collagenase IV (Servicebio), 1 mg/mL dispase (Yeasen), and 50 μg/mL DNase I (Solarbio, Beijing, China) for 30–40 min at 37°C with shaking, with vortexing every 10 min. The suspension was filtered through a 70-μm strainer and centrifuged. Cells were resuspended in 40% Percoll, layered over 80% Percoll (Yeasen). Density gradient centrifugation was performed at 1,100 × *g* for 20 min at room temperature with no brake. LPLs were collected from the interface, washed, and used for further analysis.

### IHC staining

Colon paraffin sections were baked at 65°C, deparaffinized, and rehydrated. Heat-mediated antigen retrieval was performed. Staining was performed using a commercial IHC kit (Zhong Shan Golden Bridge Biological Technology, Beijing, China). The sections were then permeabilized with 0.5% Triton X-100, and endogenous peroxidase activity was quenched with hydrogen peroxide. After blocking with 10% goat serum, the sections were incubated with specific primary antibody working solutions (diluted 1:100–300) overnight at 4°C. The sections were subsequently incubated with an HRP-conjugated secondary antibody at room temperature. Color development was initiated with a 3,3’-diaminobenzidine (DAB) substrate, and the reaction was stopped upon optimal signal intensity under microscopic monitoring. The nuclei were counterstained with hematoxylin, followed by dehydration and mounting with resin. Quantitative analysis of positive signals was performed using ImageJ software.

### Statistical analysis

All statistical analyses were performed using GraphPad Prism 8.0. Data distribution was first assessed for normality using the Shapiro-Wilk test. For comparisons between two independent groups that passed normality, the unpaired two-tailed Student’s *t* test was used. For comparisons between two independent groups with small sample sizes or when normality was violated, the Mann-Whitney U test was used; results are presented as the median and 95% CI of the median difference. For comparisons among three or more independent groups that passed normality and equal-variance tests, one-way ANOVA followed by Tukey’s post hoc test was performed. For comparisons among three or more independent groups with small sample sizes or non-normal distribution, the Kruskal-Wallis test followed by Dunn’s post hoc test with Bonferroni correction was used. For paired samples, the Wilcoxon signed-rank test was applied, and the median difference with 87.5% CI was reported. For comparisons among multiple treatment groups over time, unpaired Student’s *t* tests with Bonferroni correction for multiple comparisons were performed. For correlation analyses, Pearson or Spearman correlation was used as appropriate. Data are presented as the mean ± SD unless otherwise noted; for small-sample comparisons, data are shown as scatterplots with median and CI as indicated in the figure legends. *p* < 0.05 was considered to indicate statistical significance.

## Data and code availability

The data that support the findings of this study are available upon reasonable request.

## Acknowledgments

This work was supported by the 10.13039/501100001809National Natural Science Foundation of China (grant no. 81800493), the Teaching Research Project of the Huazhong University of Science and Technology (no. 2021148), and the Key Scientific Research Project of Hunan Provincial Department of Education (23A0663).

## Author contributions

Y.W. conducted the experiments. X.Y. analyzed the data. Y.W. and X.Z. wrote the original draft. H.W. reviewed and edited the manuscript. D.T. provided clinical samples and supervised the project. G.W. conceived and designed the study and acquired funding. All authors reviewed and approved the final manuscript.

## Declaration of interests

The authors declare no competing interests.

## Declaration of generative AI and AI-assisted technologies in the manuscript preparation process

During revision of this manuscript, an AI-assisted language tool was used to translate selected Chinese passages into English and to improve grammar and academic expression. The tool was not used to generate figures, process images, analyze data, or determine the scientific conclusions of the review. The authors reviewed and edited all translated content and take full responsibility for the accuracy, integrity, and final wording of the manuscript.

## Supplemental information

Document S1. Figures S1–S7 and Table S1

Document S2. Article plus supplemental information
