# Supplementary material for: Targeting miR-21 ameliorates colitis by restoring Th17/Treg balance through direct inhibition of Smad7/Ski
Source: Mol Ther Nucleic Acids. 2026 Sep 29;37(4):103120. doi: 10.1016/j.omtn.2026.103120 (PMC13691311; doi:10.1016/j.omtn.2026.103120)
Supplement: Supplementary file 1 [file mmc1.pdf]

OMTN, Volume *37*

Supplemental information

Targeting miR-21 ameliorates colitis by restoring Th17/Treg balance through direct inhibition of Smad7/Ski

Yue Wen, Xia Yi, Xiaowen Zhu, Han Wang, Dean Tian, and Ge Wang

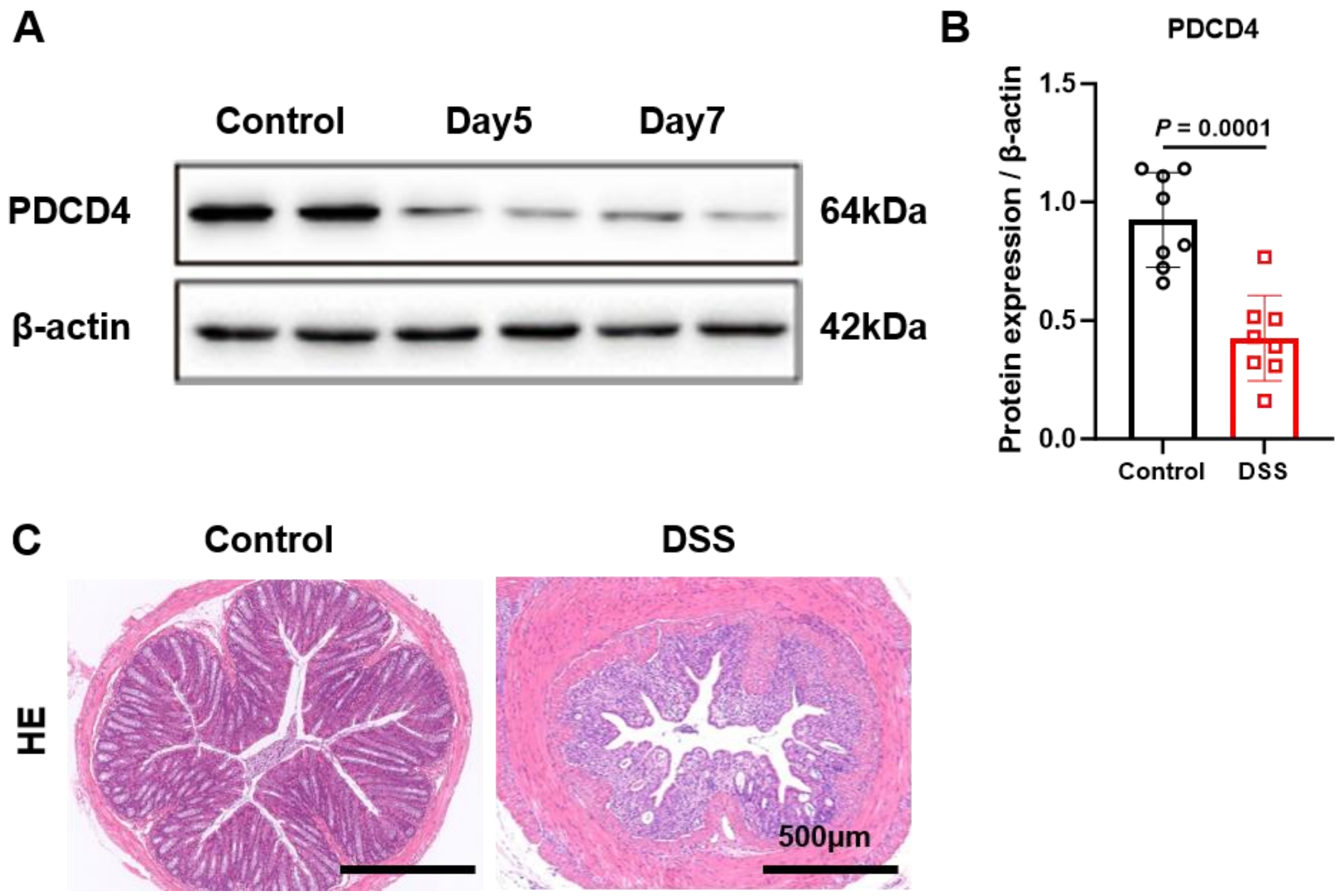

Figure S1. Expression of PDCD4 in DSS-induced acute colitis mice. (A) Representative immunoblot of PDCD4 protein expression in mouse colon tissues. (B) Quantitative analysis of PDCD4 protein levels (n = 8). Data were analyzed by unpaired Student's t-test. (C) Representative H&E-stained images of colon sections (10× magnification). Scale bar = 500 μm. Data are presented as mean ± SD.

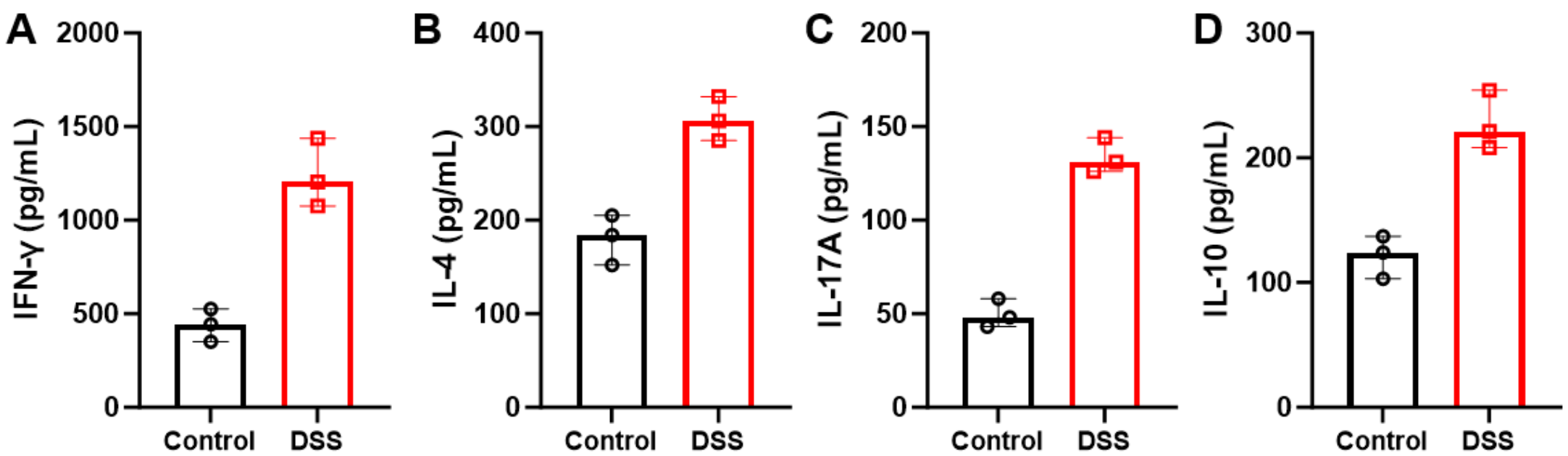

Figure S2. Altered inflammatory cytokine levels in colons of DSS-induced acute colitis mice. (A-D) ELISA analysis of protein expression levels for IFN-γ, IL-4, IL-17A, and IL-10 in mouse colon tissues (n = 3). Data were analyzed by Mann-Whitney U test, and results are presented as scatter plots with median and 95% confidence interval.

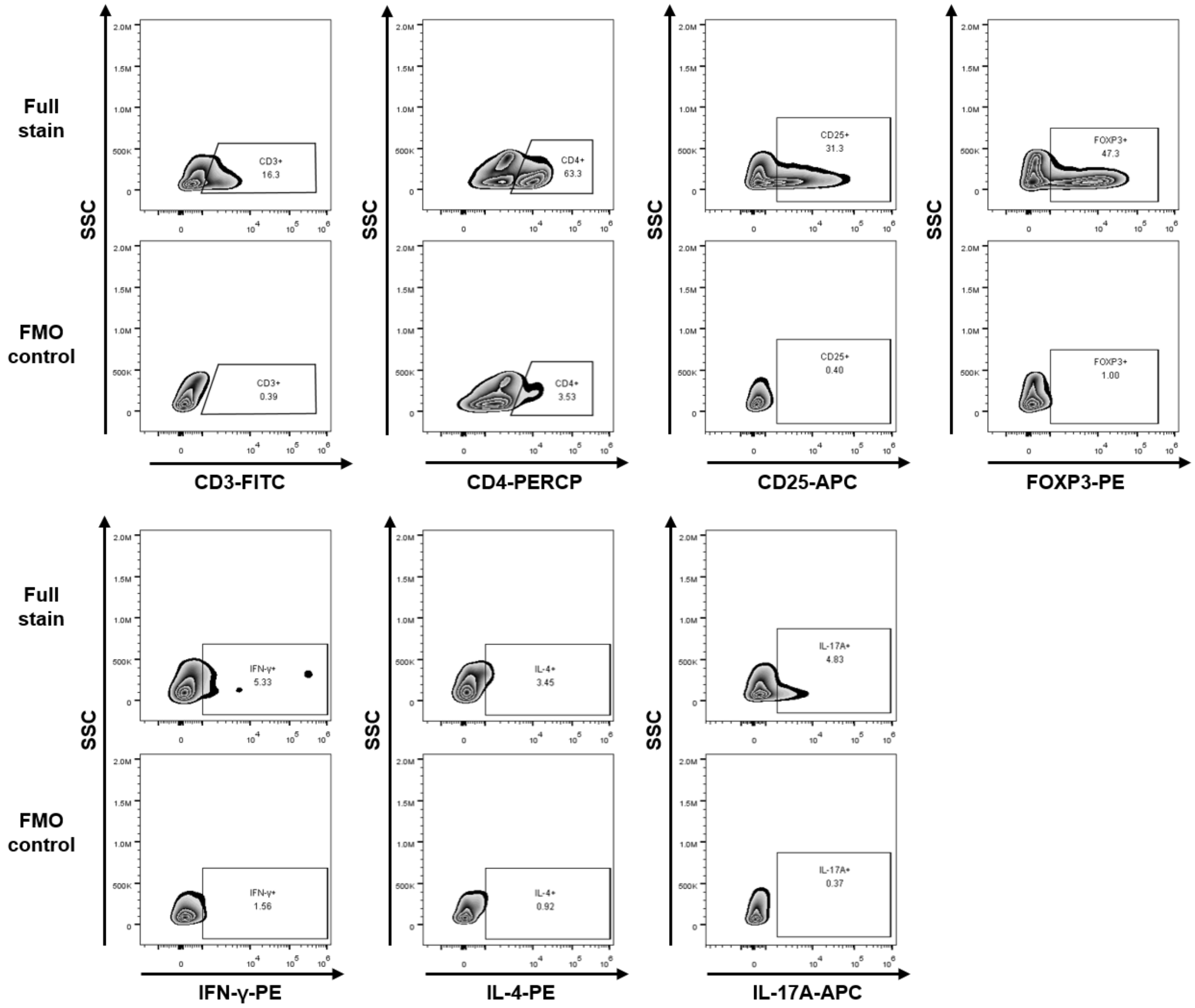

Figure S3. Fluorescence-minus-one (FMO) controls for flow cytometry gating. Representative plots showing full staining (CD3-FITC, CD4-PerCP, CD25-APC, Foxp3-PE, IFN-γ-PE, IL-4-PE and IL-17A-APC) and the corresponding FMO controls for each fluorescence channel, in which the indicated antibody was omitted.

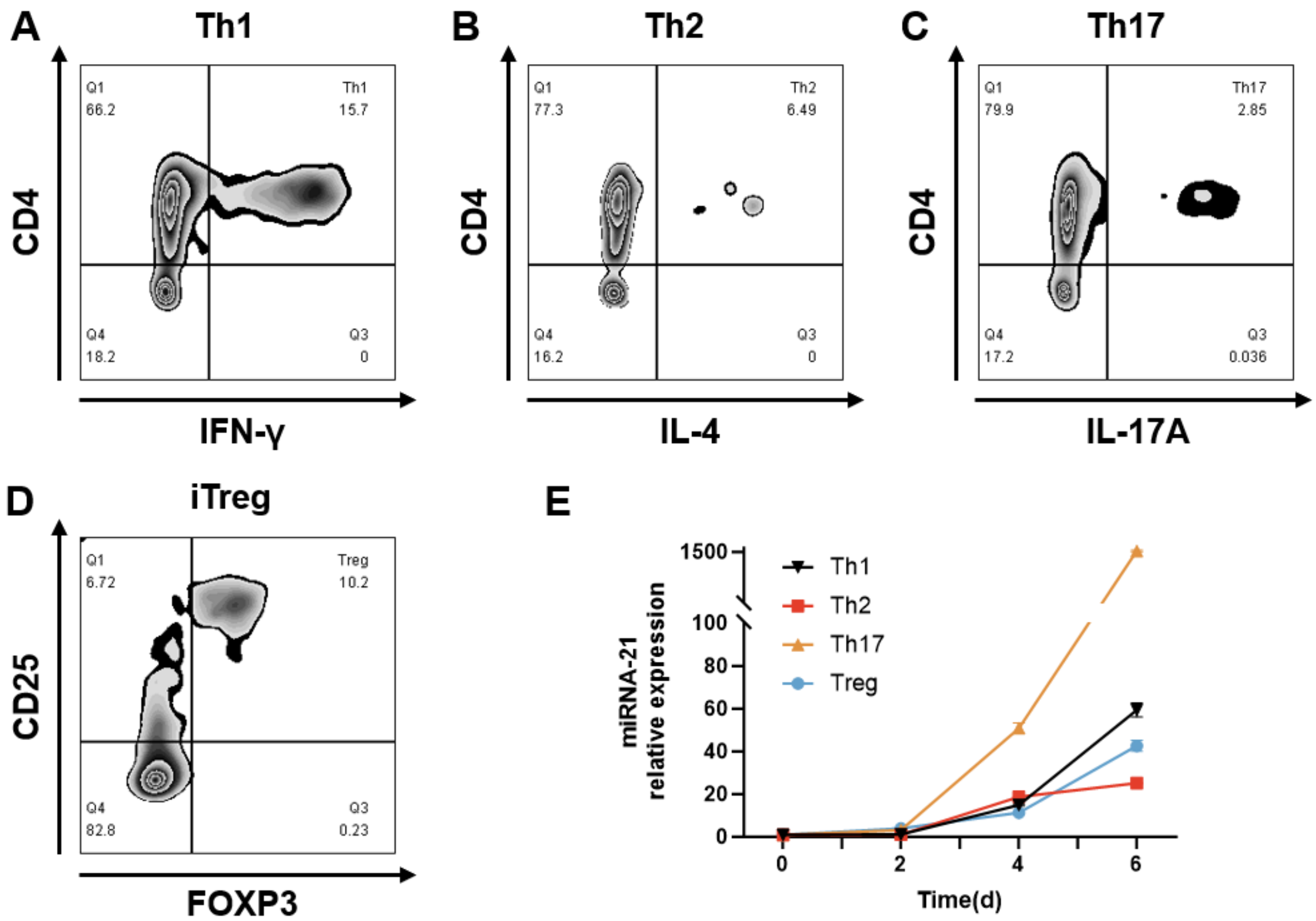

Figure S4. Elevated miR-21 expression during Th17 cell differentiation. (A-D) Percentages of polarized Th1, Th2, Th17, and Treg cells derived from naïve CD4^{+} T cells, assessed by flow cytometry. (E) Relative miR-21 expression levels at indicated differentiation time points in Th1-, Th2-, Th17-, and Treg-polarized cells, measured by RT-qPCR (n=3).

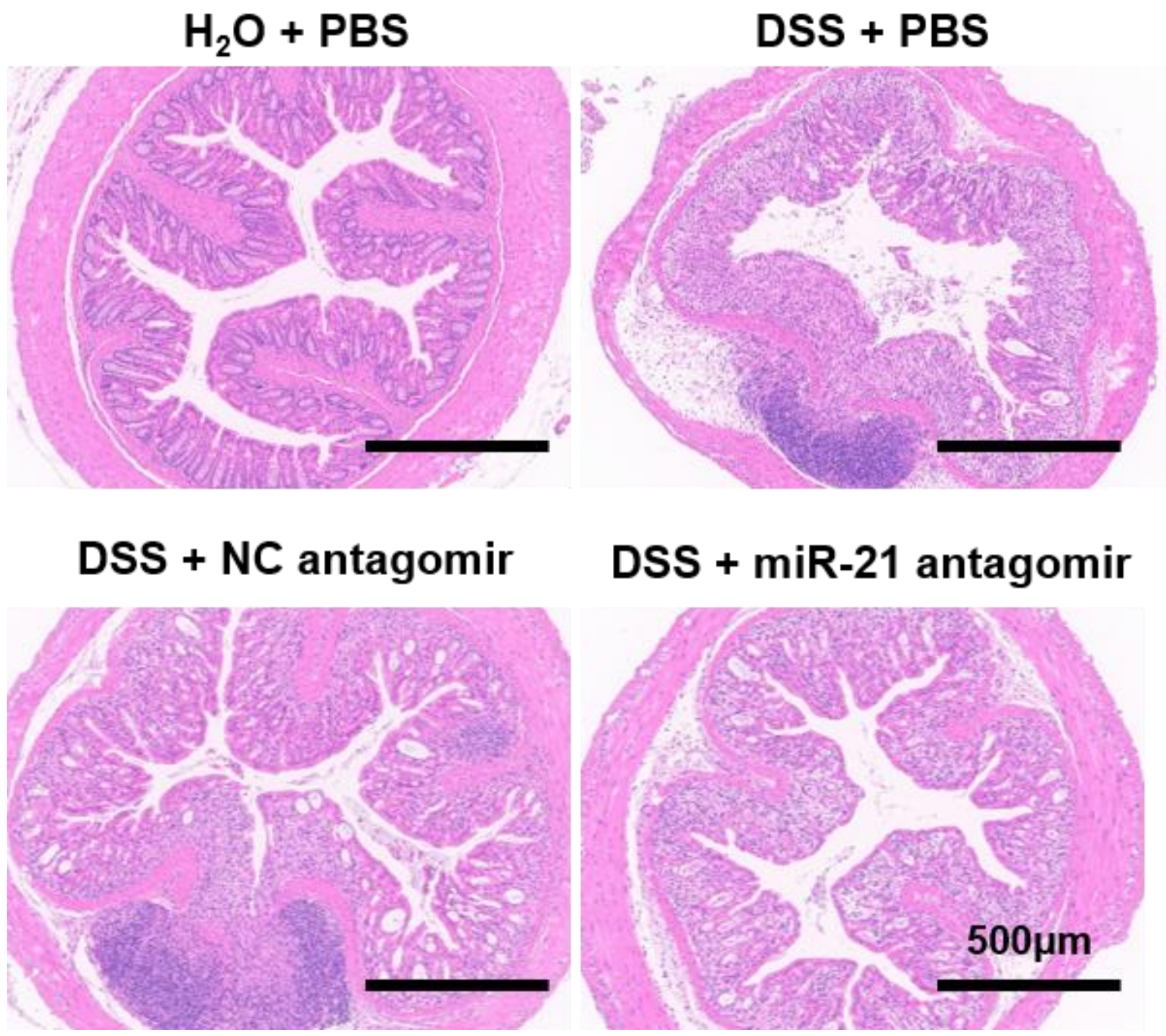

Figure S5. Representative H&E-stained images of colon sections (10× magnification).

Scale bar = 500 μm.

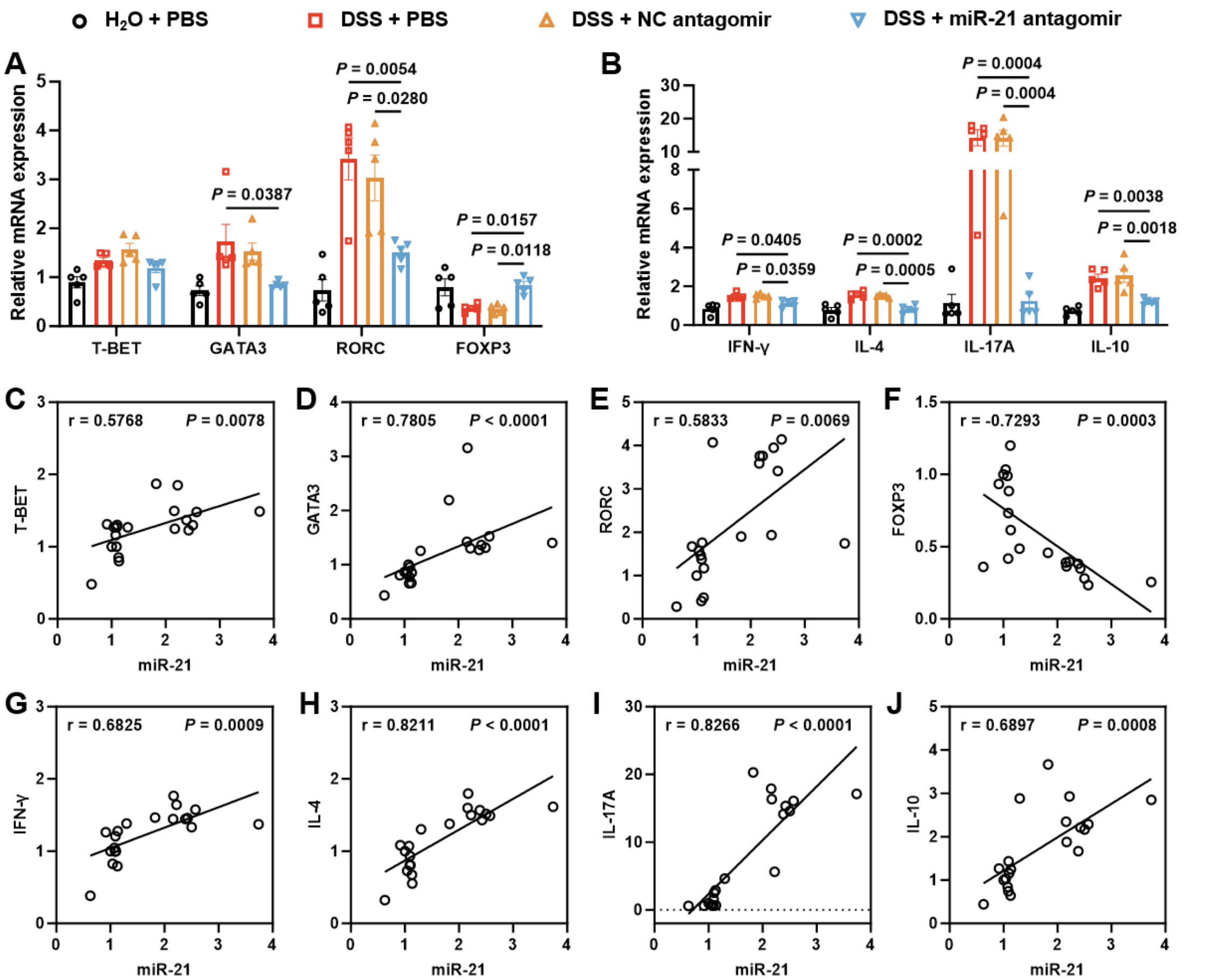

Figure S6. mRNA expression and correlation with miR-21 in colonic lamina propria lymphocytes. (A) mRNA expression levels of T-bet, GATA3, RORγt, and Foxp3 in LPLs (n = 5). (B) mRNA expression levels of IFN-γ, IL-4, IL-17A, and IL-10 in LPLs (n = 5). (C-J) Correlation analysis between miR-21 level and the mRNA levels of T-bet, GATA3, RORγt, Foxp3, IFN-γ, IL-4, IL-17A, and IL-10 (n = 20). Data in (A, B) were analyzed by one-way ANOVA followed by Tukey's post hoc test. Data in (C-J) were analyzed by correlation analysis. Data are presented as mean ± SD.

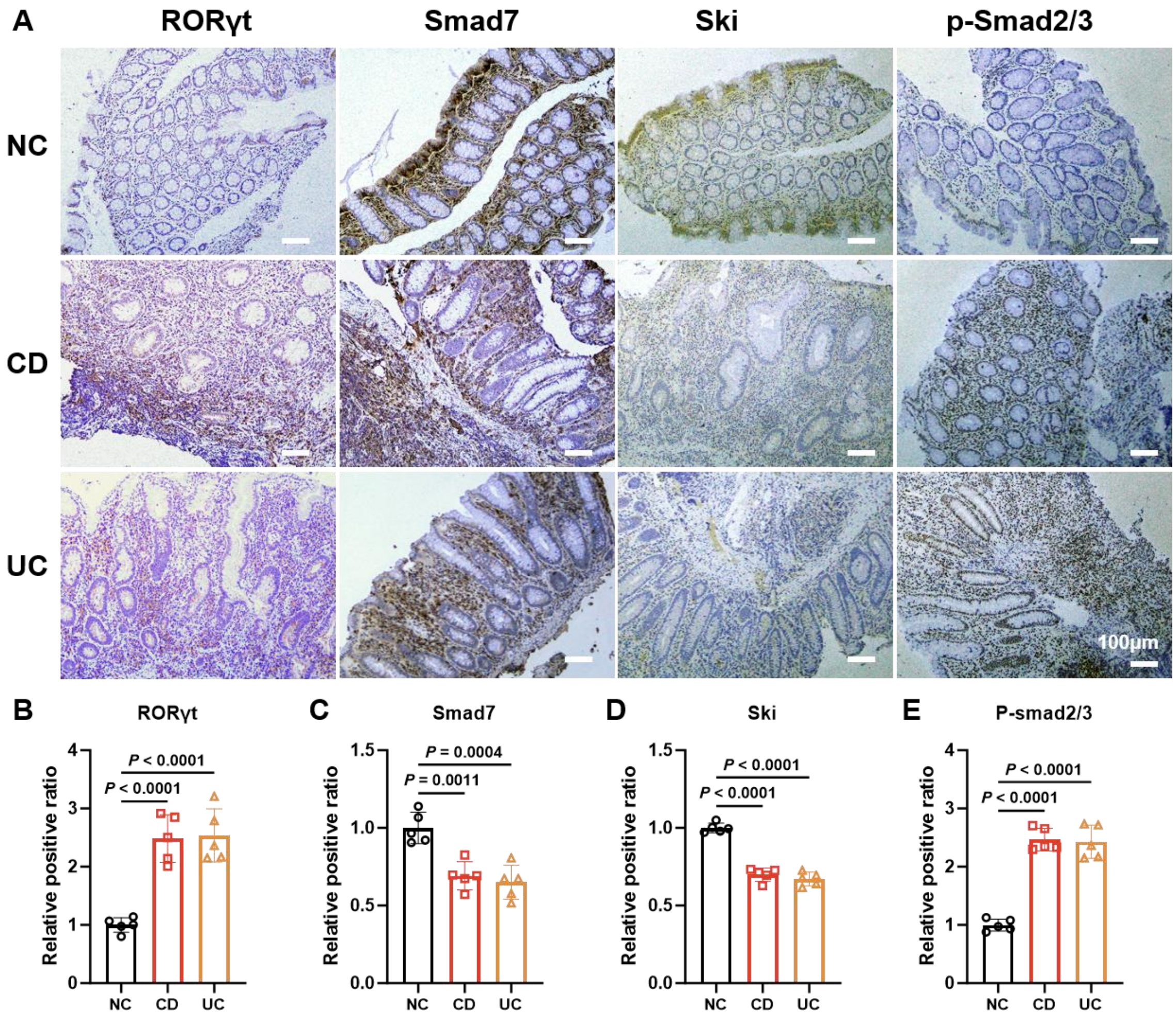

Figure S7. Immunohistochemical analysis of RORγt, Smad7, Ski, and p-Smad2/3 in human colonic biopsies. (A) Representative IHC staining of RORγt, Smad7, Ski, and p-Smad2/3 in colonic mucosa from normal controls (polyp patients), active CD, and active UC patients. Scale bar = 100 μm. (B-E) Semi-quantitative scoring of RORγt (B), Smad7 (C), Ski (D), and p-Smad2/3 (E) expression levels (n = 5). Data in (B-E) were analyzed by one-way ANOVA followed by Tukey's post hoc test. Data are presented as mean ± SD.

Table S1. Primer sequence

Primer name	Sequence（5' → 3'）
U6-F	CTCGCTTCGGCAGCACA
U6-R	AACGCTTCACGAATTTGCGT
miR-21 RT primer	GTCGTATCCAGTGCAGGGTCCGAGGTATTCGCAC TGGATACGACTCAACA
miR-21-F	AGTGCAGGGTCCGAGGTATT
miR-21-R	GCGCGTAGCTTATCAGACTGA
mmu-GAPDH-F	TGACCTCAACTACATGGTCTACA
mmu-GAPDH-R	CTTCCCATTCTCGGCCTTG
mmu-18s-F	GTAACCCGTTGAACCCCATT
mmu-18s-R	CCATCCAATCGGTAGTAGCG
mmu-IFN-γ-F	GCGCCAAGCATTCAATGAGC
mmu-IFN-γ-R	ATCTCTTCCCCACCCCGAAT
mmu-IL-4-F	GGTCTCAACCCCCAGCTAGT
mmu-IL-4-R	GCCGATGATCTCTCTCAAGTGAT
mmu-IL-17A-F	TCAGCGTGTCCAAACACTGAG
mmu-IL-17A-R	CGCCAAGGGAGTTAAAGACTT
mmu-IL-10-F	CTTACTGACTGGCATGAGGATCA
mmu-IL-10-R	GCAGCTCTAGGAGCATGTGG
mmu-T-BET-F	AGTGATTGGTTGGAGAGGAAG
mmu-T-BET-R	GGGACACTCCTGTCGTATTTC
mmu-GATA3-F	CTCGGCCATTCGTACATGGAA
mmu-GATA3-R	GGATACCTCTGCACCGTAGC

mmu-RORC-F	TCCACTACGGGGTTATCACCT
mmu-RORC-R	AGTAGGCCACATTACACTGCT
mmu-Foxp3-F	ACCATTGGTTTACTCGCATGT
mmu-Foxp3-R	TCCACTCGCACAAAGCACTT
mmu-SMAD7-F	GGGCTTTCAGATTCCCAACTT
mmu-SMAD7-R	AGGGCTCTTGGACACAGTAGA
mmu-SKI-F	CAAAACAGACGACACTTCCTCA
mmu-SKI-R	CAGCCGAGGCTCTTATTGGAG
mmu-TGFBR2-F	AACATGGAAGAGTGCAACGAT
mmu-TGFBR2-R	CGTCACTTGGATAATGACCAACA
mmu-TGFBI-F	TGTCCTGGATATGAAAAGGTCCC
mmu-TGFBI-R	GGTGGTCGATCCCACAACTC
mmu-WWP1-F	TGGGAGGTTCCAATATCCTGG
mmu-WWP1-R	ACGCCATTCTGTCATTAAACCA
mmu-STAT3-F	AATATAGCCGATTCCTGCAAGAG
mmu-STAT3-R	TGGCTTCTCAAGATACCTGCTC
